# Effects of Hydrogen Peroxide on Slow- and Fast-Growing NIH/3T3-Derived Cultures: Nuclear and Cytoplasmic Aspects Related to Senescence and Transformation

**DOI:** 10.3390/cells14161268

**Published:** 2025-08-16

**Authors:** Alessandra Spano, Luigi Sciola

**Affiliations:** Department of Biomedical Sciences, University of Sassari, Via Muroni 25, I-07100 Sassari, Italy; alspano@uniss.it

**Keywords:** proliferation, senescence, transformation, redox status, mitochondria, lysosomes

## Abstract

Cellular senescence can occur with similar phenotypes in normal cells, during aging, and in tumor cells, spontaneously or after cytostasis. The fall or increase in proliferative activity are key aspects of the respective conditions, in which the levels of reactive oxygen species can vary, affecting the cellular redox homeostasis. This work aimed to study the relationships between senescence and transformation by comparing cells with different proliferative activities and phenotypes attributable to transformation (NIHs cultures) or senescence (NIHv cultures), before and after incubation with hydrogen peroxide. Both cultures were derived from the NIH/3T3 cell line, which was used here as a reference (NIHb), after the serum starvation. Our experimental model can be representative of the heterogeneity of cell subpopulations, with different degrees of transformation and senescence, found in some tumors. The characterization of the functional properties of NIHb, NIHs, and NIHv cells was performed by a morphocytometric analysis of the cell cycle progression, mitochondrial and lysosomal content/activity, and superoxide anion production. The efficiency of the lysosomal compartment was also assessed by estimating the autophagic activity and measuring lipofuscin autofluorescence. Comparisons of nuclear and cytoplasmic parameters before and after the incubation with hydrogen peroxide revealed differences in the expression and modulation of cellular senescence patterns. The treatment effects were very limited in the NIHb culture; the senescence condition was essentially maintained in the NIHv cells, while the most relevant changes were found in the NIHs cells. In the latter, the acquisition of the senescent phenotype, also demonstrated by the positivity of SA-β-galactosidase, was correlated with a decrease in proliferative activity and a change in the content/activity of the mitochondria and lysosomes, which showed similarities with the basal senescence conditions of NIHv cells. In NIHs cells, increased autophagy events and lipofuscin accumulation also indicate the establishment of cytoplasmic dynamics typical of senescence. The variable responses to hydrogen peroxide, besides depending on the different basal cytokinetic activity of the cultures examined, appeared to be related to the specific cell redox state resulting from the balance between endogenous ROS and those produced after treatment. Especially in NIHs cells, the slowing down of the cell cycle was linked to dynamic interconnections between the mitochondrial and lysosomal compartments. This would indicate that transformed cells, such as NIHs, may express morpho-functional aspects and markers typical of cellular senescence, as a consequence of the modulation of their redox state.

## 1. Introduction

More than 60 years ago, Hayflick and Moorhead used the term “cellular senescence” referring to studies performed on human cell strains that showed a growth arrest after a defined number of in vitro subcultures [1]. Subsequent studies have shown that senescent cells can accumulate in different tissues during aging in mammals [2]. In these organisms, due to the collapse of the proliferative capacity, senescent cells can prevent tissue renewal, playing a critical role in tissue homeostasis. The implications of cellular senescence, in response to stress conditions associated with aging organisms, are also documented in the onset and evolution of cancer [3,4]; however, many aspects in this context need to be clarified. In the last decade, especially in recent years, several lines of experimental evidence have supported the view that tumor cells may undergo senescence not only after chemotherapy [5] or a treatment with ionizing radiation [6] but also spontaneously [7]. The triggering of senescence may depend on the nature of the stress induced and its intensity, as well as the phenotypic characteristics of the cell type involved. The permanent reduction in the proliferative capacity following the induction of senescence in tumor cells appears to be the basis for the development of a new alternative therapeutic strategy to cytostasis induced by drastic cytotoxic treatments [8]. However, in contrast to the considerable amount of information on premature senescence induced by different mechanisms in normal cells, the processes underlying the onset of senescence in tumor cells need to be fully elucidated. Among the latter, the analysis of the phenotypes of those that spontaneously become senescent has shown that they express features similar to those of normal senescent cells [7]. However, tumor cell senescence is a phenomenon with complex bases. In various cellular models, previous studies have shown that the inhibition of proliferative activity, which is linked to the manifestation of the senescent phenotype, occurs through the long-term activation of the CDK inhibitor p21 [9,10]. Besides being reflected in cell cycle arrest, p21 also induces functional abnormalities at the mitochondrial level with an increase in reactive oxygen species (ROS) [9,11]. Although a decisive role may also be played by ROS in cellular senescence, the cause-and-effect relationships between their production and some manifestations of the process are not entirely clear. The ineffectiveness against cell proliferation and the poor prognosis of some classical therapeutic protocols may be based on the heterogeneity between different cell types within the same tumor. In these cases, the different resistances to chemotherapeutic agents could be influenced by the variable reactivity of the cells toward the ROS levels generated during certain treatments. In general, different oxidative agents can alter the concentration level of intracellular oxidants, with varying effects on cell proliferation. Studies performed so far have indicated that a treatment with hydrogen peroxide can induce cellular senescence in normal cells [11], while the effects on tumor cells appeared more variable. In the latter, the greater proliferative activity, related to the higher intracellular concentration of hydrogen peroxide, than in normal cells, could increase their vulnerability to oxidative stress [12,13]. In recent years, various studies have indicated the importance of the relationship between oxidative stress and cellular senescence in tumor development. The complexity and variability that emerged in some cases appeared to be related to the stage of the tumor progression and to the phenotypic diversity of the cells in their microenvironment. In this condition of heterogeneity, subpopulations of cells with different degrees of transformation or senescent status may coexist. On the other hand, in certain situations, transformed and non-transformed senescent cells may become capable of expressing tumor-promoting properties [14]. These processes can be influenced by the level of intracellular ROS, which at low concentrations, can act as signal molecules, promoting heterogeneity and cell transformation, whereas at higher concentrations they can induce genotoxic or proapoptotic effects [11,15]. Given these premises, the acquisition of knowledge in this field is still fundamental and can allow us to interpret the complexity of the senescence phenomenon and how the programs of its expression can be related to the specific characteristics of the cellular phenotypes involved. In this context, useful information can be obtained with an in vitro experimental approach based on the induction of oxidative stress in a comparative system represented by different cultures in whose cells certain characteristics typical of the senescent or transformed phenotype are alternately identifiable. Over the past few years, we have developed an in vitro cell model capable of responding at least in part to these characteristics. It consists of two cultures that have shown, over time, the prevalence of cells with morpho-functional aspects attributable to cell transformation or senescence. The cultures, named NIHs and NIHv, are derived from a single cell line named NIH/3T3 (referred to here as NIHb), which is subjected to prolonged serum deprivation and the selection of cells with different phenotypic features [16]. As shown in previous work, critical culture conditions induced by serum deprivation can lead to increased oxidants at the cellular level. The greater number of spontaneous mutations detected in cultures could therefore be the result of oxidative stress, which is also caused by the lack of antioxidants in the serum [17]. In our opinion, the experimental model used in the present work may be at least partly representative of, or present similarities with, the conditions of cellular heterogeneity that may characterize certain tumors. On the basis of the above, the present study seeks to contribute, by means of morphological and cytometric approaches, to the interpretation of certain cellular aspects inherent to the complexity of the interrelationships linking senescence to cell transformation processes. The heterogeneity of the phenotypes of the cells constituting the NIHs and NIHv cultures was analyzed before and after the incubation with hydrogen peroxide. The original NIH3T3 line (NIHb) was used as a normal reference culture. In the first phase of the work, we dealt with the characterization of the cell model from morphological and cytokinetic points of view. The cell cycle analysis of the three cultures under study was performed by means of the flow cytometry of the DNA content and the incorporation of Bromodeoxyuridine. We subsequently performed preliminary analyses for the treatment of cultures with hydrogen peroxide to define the sublethal conditions. On the basis of these analyses, we chose a concentration of 75 μM with an incubation time of 3 h. The analysis of the responses induced by the treatment with this oxidizing agent was aimed at assessing the influence of changes in the redox state and senescence patterns expressed in cells characterized by different phenotypic properties. In addition to the effects of hydrogen peroxide on the cell cycle, we found it useful to relate changes in its progression to fundamental functional changes at the cytoplasmic level. We therefore considered the dynamics involving the mitochondria and lysosomes and the processes related to their interaction. As is well known, these organelles show significant changes, both in structure and function, during the processes of cellular transformation and senescence, partly as a function of the changes in the redox state linked to these biological events. The flow cytometry/microfluorimetry methods applied, in addition to detecting the production level of the ROS superoxide anion, made it possible to determine parameters such as the mitochondrial and lysosomal content/activity. The progressive dysfunction of the mitochondria may not be compensated for by their removal, due to abnormalities in the activity of lysosomes, which should instead play a fundamental role in this process [18]. The occurrence of lysosomal dysfunctions may lead to the deterioration of not only the mitochondrial turnover but also a consequent increase in ROS, which also negatively affects the structure and function of the lysosomes themselves [19]. To obtain information in this context, we evaluated the cytoplasmic presence of lipofuscin inclusions and their relationships with the expression of autophagic events, which may be a possible cause of the progressive accumulation of the undigested lipofuscin material. As a further indicator of lysosomal functionality, the SA-β-galactosidase (SA-β-gal) activity, detected at a pH of 6.0, was analyzed. At this pH value, the SA-β-gal enzyme usually shows an increased activity in senescent cells: this is a sign of altered lysosomal acidification [20].

Together with the analysis of the proliferative aspects, the application of these methodologies allowed us to obtain a comprehensive overview of the cytokinetic and cytoplasmic characteristics of NIHb, NIHs, and NIHv cultures compared both under basal conditions and after the treatment with hydrogen peroxide.

## 2. Materials and Methods

### 2.1. Experimental Model

In the present work, an in vitro cell model consisting of two cell cultures derived from the NIH/3T3 mouse embryo fibroblast line was used. These cultures were obtained after the isolation and propagation of cells with different morpho-functional characteristics related to phenotypes attributable to transformation (NIHs) and cellular senescence (NIHv). Some details of this experimental model are given below. The cells from the original NIH/3T3 line were subjected to prolonged (7–10 days) serum deprivation (culture medium containing 0.5% fetal calf serum). The withdrawal of serum from the culture medium caused massive cell detachment and the death of almost all the cells in the culture. When the serum was restored, some of the surviving cells re-entered the cell cycle, restoring proliferative activity. The entire culture also showed the presence of heterogeneous subpopulations: the largest cell fraction showed a lower capacity for adhesion to the growth substrate (compared with the original NIH/3T3 line here called NIHb) and a tendency to form foci of actively proliferating fibroblasts that were arranged in multiple layers. Cells isolated from these areas (later named NIHs) can grow in suspension and give rise to aggregates in the form of spheroids. Compared with the original NIH/3T3 cell line (NIHb), the appearance of polyploid cells with DNA content values between 4c and 24c was detected in the later propagation phases of the NIHs culture [16]. In NIHs cultures, the presence of polyploid cells can express genetic instability and susceptibility to cell transformation. After serum was restored in the culture medium, a minority fraction of the cells (subsequently named NIHv) showed marked adhesion to the growth substrate and an enlarged and flattened appearance. These morphological features appeared to be correlated with lower proliferative activity (compared with that of the original NIH/3T3 cell line) and the accumulation of autofluorescent lipofuscin inclusions in the cytoplasm. Overall, these cytological features can be considered indicative of a cellular senescent phenotype.

### 2.2. Selection and Enrichment of NIHs and NIHv Cells

The cultures obtained after serum withdrawal were heterogeneous and consisted of cells with different adhesive and proliferative properties, which were used for the selection and enrichment of NIHs and NIHv cells. After isolation by aspiration, the spheroids originating from the foci of active cell proliferation were transferred into culture flasks. The cells originating from spheroids, after adhesion to the growth substrate, were dissociated by trypsinization and subsequently propagated, resulting in NIHs culture.

To obtain cultures consisting of cells with an enlarged/flattened appearance and marked adhesion to the growth substrate, the heterogeneous cultures obtained after serum starvation were subjected to trypsinization but for short times (4 min at 37 °C). These conditions resulted in the prevailing detachment of poorly adherent and actively proliferating cells and retention in the growth substrate of those with greater adhesive capacity. To obtain enriched cultures of these strongly adherent cells, here called NIHv, this trypsinization procedure was repeated 3 to 5 times. In this way, almost complete detachment of the poorly adherent, actively proliferating cells was achieved during the subsequent propagation phases. After adhesion, the NIHv cells showed slow proliferation only in some areas of the growth substrate. For the different cultures used here, the following times were necessary to detach the cells from the growth substrate: NIHb cells: 7 min; NIHs cells: 4 min; and NIHv cells: 12 min.

### 2.3. Cell Cultures

NIHb, NIHs, and NIHv cells were cultured in Dulbecco’s modified Eagle’s medium (Sigma-Aldrich, Saint-Louis, MO, USA) supplemented with 10% fetal calf serum (Gibco, ThermoFisher, Waltham, MA, USA), 2 mM L-glutamine (Gibco, ThermoFisher, Waltham, MA, USA), 100 units/mL penicillin (Gibco, ThermoFisher, Waltham, MA, USA), and 100 μg/mL streptomycin (Gibco, ThermoFisher, Waltham, MA, USA) at 37 °C in a humidified atmosphere containing 5% CO_2_. For propagation, the different cultures were incubated with 0.25% trypsin (Gibco, ThermoFisher, Waltham, MA, USA) in calcium- and magnesium-free Dulbecco’s phosphate-buffered saline (PBS) (Gibco, ThermoFisher, Waltham, MA, USA) at 37 °C. Cultures were analyzed as sub-confluent monolayer under all the experimental conditions.

### 2.4. Hydrogen Peroxide Treatment

The cell cultures were incubated at 37 °C with 75 µM hydrogen peroxide (H_2_O_2_) (Sigma-Aldrich, Saint-Louis, MO, USA) for 3 h in complete medium. This treatment was defined on the basis of a series of preliminary analyses that allowed us to establish sublethal conditions. At the end of the incubation, after being washed twice with PBS (Sigma-Aldrich, Saint-Louis, MO, USA), the cells were fixed or processed immediately for analysis under cell viability conditions.

### 2.5. Microscopy and Photographic Documentation

Photographic documentation was obtained using a Zeiss Axiovert 40C inverted phase contrast microscope (Carl Zeiss Jena GmbH, Jena, Germany) in a conventional mode with adequate illumination to obtain an interference contrast effect. A Nikon Eclipse 600 microscope (Nikon, Kanagawa, Japan) equipped for epifluorescence observation of fluorochromized samples was used.

### 2.6. Cell Cycle Analysis

The different cultures were grown in 25 cm^2^ flasks until a cell confluence of 80% was reached. To obtain preliminary information on the proliferative activity of the cells constituting the three different cultures under study, the DNA content was analyzed by flow cytometry after propidium iodide (PI) (Sigma-Aldrich, Saint-Louis, MO, USA) fluorochromization. To obtain more detailed information on the percentage of cells in the S phase, PI staining was subsequently combined with immunoreaction using FITC-conjugated anti-5-Bromo-2′-deoxyUridine (BrdU) antibodies (Sigma-Aldrich, Saint-Louis, MO, USA). The cells were previously incubated with BrdU to achieve incorporation into the DNA during duplication.

#### 2.6.1. Determination of DNA Content by Means of Propidium Iodide Staining

After washing the untreated cells and those treated with hydrogen peroxide, the monolayers were incubated with a trypsin/EDTA mixture (Gibco, ThermoFisher, Waltham, MA, USA) to detach the cells, which were harvested by centrifugation at 200× *g* for 10 min. The pellets were resuspended in PBS (Sigma-Aldrich, Saint-Louis, MO, USA), and the cells were fixed with 4% paraformaldehyde (Sigma-Aldrich, Saint-Louis, MO, USA) for 20 min and then permeabilized with 0.5% Triton X-100 (Sigma-Aldrich, Saint-Louis, MO, USA) for 15 min. Finally, the cells were centrifuged and resuspended in a PBS solution containing 1% BSA (Sigma-Aldrich, Saint-Louis, MO, USA), 10 μg/mL propidium iodide (Sigma-Aldrich, Saint-Louis, MO, USA), and 0.25 μg/mL RNase A (Sigma-Aldrich, Saint-Louis, MO, USA) and incubated in the dark at 37 °C for 30 min. Before flow cytometric analysis, the cells were passed through Nytex filters to remove aggregates. Flow cytometry data were plotted as DNA content histograms (PI-fluorescence). The percentage of cells in distinct phases of the cell cycle was calculated after deconvolution of the DNA content frequency histograms using FlowMax software 2.3 (Partec GmbH, Münster, Germany). 

#### 2.6.2. Evaluation of BrdU Uptake

Both untreated and hydrogen peroxide-treated cell monolayers were incubated at 37 °C with 10 µM BrdU (Sigma-Aldrich, Saint-Louis, MO, USA) for 30 min. At the end of incubation with BrdU, after washing with PBS (Sigma-Aldrich, Saint-Louis, MO, USA) to remove traces of medium, the cell monolayers were incubated with a trypsin/EDTA mixture (Sigma-Aldrich, Saint-Louis, MO, USA) to detach the cells which were harvested by centrifugation at 200× *g* for 10 min. Pellets were resuspended in PBS (Sigma-Aldrich, Saint-Louis, MO, USA), and the cells were fixed with 4% paraformaldehyde (Sigma-Aldrich, Saint-Louis, MO, USA) for 20 min and then permeabilized with 0.5% Triton X-100 (Sigma-Aldrich, Saint-Louis, MO, USA) for 15 min. Subsequently, the nuclear DNA was denatured with 2 N HCl (Sigma-Aldrich, Saint-Louis, MO, USA) for 30 min; neutralization was performed with 0.1 M sodium borate (Sigma-Aldrich, Saint-Louis, MO, USA) (pH 8.5). To block nonspecific binding sites, the cells were incubated with 10% normal serum for 1 h at room temperature. Washing with PBS (Sigma-Aldrich, Saint-Louis, MO, USA) and centrifugation of the cells were included among the fixation, permeabilization, denaturation, and neutralization steps.

For immunolabeling, the cells were incubated for 45 min, at room temperature, with primary mouse anti-BrdU antibodies (dilution 1:50) (Sigma-Aldrich, Saint-Louis, MO, USA). After washing and centrifugation, the cells were resuspended again and incubated for 45 min in a solution containing FITC-conjugated rabbit anti-mouse secondary antibodies (dilution 1:25) (Sigma-Aldrich, Saint-Louis, MO, USA). The negative controls included immunoreactions in which the primary antibody was omitted, and only the secondary antibody was used. Furthermore, the incubation with BrdU and the subsequent immunoreactions were preliminarily carried out on nonproliferating NIH/3T3 cultures, under conditions of serum starvation for several days to verify the presence of false positives.

For DNA fluorochromization with PI (Sigma-Aldrich, Saint-Louis, MO, USA), the same procedure was followed as for the monoparametric analysis above. Before flow cytometric analysis, the cells were passed through Nytex filters to remove aggregates.

The flow cytometric data of the double fluorochromization were represented as dot plots in which the red fluorescence (λem: 610 nm) of the PI was reported on the abscissa (linear scale), and the green fluorescence (λem: 520 nm) of the FITC-conjugated anti-BrdU was reported on the ordinate (logarithmic scale).

### 2.7. Flow Cytometric Content/Activity Analysis and Morphology Evaluation of Mitochondria

#### 2.7.1. Flow Cytometry

Changes in the mitochondrial compartment were estimated using the DiOC6 (Diexyoxyarbocyanine iodide) probe (Sigma-Aldrich, Saint-Louis, MO, USA). Morphological analysis was performed by fluorescence microscopy, and quantitative analysis was performed by flow cytometry. The DiOC6 probe is a lipophilic cationic fluorochrome that readily crosses the plasma membrane and mitochondrial membranes. The amount of fluorochrome accumulated in the mitochondrial matrix is related mainly to the magnitude of the transmembrane potential (ΔΨm) and thus to the functional activity of the organelle. However, the fluorescence emitted by the cells can also be influenced by other parameters, such as the size and shape of the cell or the density of the mitochondria in the cytoplasm (mitochondrial mass), as well as their structure (ovoid or elongated or multibranched shape). For these reasons, we have indicated the fluorescence of DiOC6, measured cytometrically in the cells of the different cultures, as mitochondrial content/activity. In this work, fluorochromization with the DiOC6 probe (Sigma-Aldrich, Saint-Louis, MO, USA) was performed under cell viability conditions in the presence of PI. The latter fluorochrome is usually excluded from viable cells with an intact plasma membrane, so its uptake level is determined by the progressive loss of selective permeability properties. In the case of plasma membrane alterations, the fluorochrome PI (Sigma-Aldrich, Saint-Louis, MO, USA), which penetrates the cell interior, accumulates mainly in the nucleus where it emits red fluorescence. Through microscopy, it is therefore possible to morphologically correlate this condition with the state of the mitochondrial compartment. From a cytometric point of view, the intensities of the green fluorescence of DiOC6 and the red fluorescence of PI can be measured separately, obtaining frequency histograms, or simultaneously, to obtain dot plots. In the latter graphs, the correlation between the green and red fluorescence signals allowed us to identify cellular subpopulations in which mitochondrial content/activity can be related to the functional state of the plasma membrane. For double staining, the fluorochromes were added directly to the culture medium in multi-well plates, in which the cells adhere to coverslips, or in cell suspension tubes. The cells were subjected to fluorescence microscopy or flow cytometric analysis, respectively. The two fluorochromes were added directly into the culture medium at concentrations of 5 μg/mL (PI) and 0.5 μg/mL (DiOC6). The cells were incubated with both fluorochromes at 37 °C for 20 min. At the end of the staining, the cells were washed in PBS (Sigma-Aldrich, Saint-Louis, MO, USA) and observed under fluorescence microscope or subjected to flow cytometric analysis in the presence of the culture medium.

For flow cytometry procedures, the cell suspensions obtained after detachment and centrifugation, following double staining, were passed through Nytex filters, to remove the aggregates, and then adjusted to a density of 1 × 10^6^ cells/mL culture medium. The samples were immediately analyzed, for the quantitative determination of individual cellular parameters, with a Partec Cyflow^®^ Space flow cytometer equipped with an argon laser that excited DiOC6 and PI at 488 nm. The green fluorescence of DiOC6 was recorded on FL1 (515–520 nm band pass filter), whereas the red fluorescence of PI was measured on FL2 (600 nm long pass filter). Cytometric data were analyzed with FlowMax software (Partec, Germany). Fluorescence measurements of DiOC6, in monoparametric mode, were represented as intensity frequency histograms and expressed numerically as mean values (M) relative to any cellular fractions present. In the same graphs, the percentage values (A) obtained from the areas subtended by the frequency curves are also shown.

The biparametric fluorescence measurements of DiOC6/PI are represented on a logarithmic scale as dot plots in which the green fluorescence (λem: 520 nm) of DiOC6 is shown on the abscissa, and the red fluorescence (λem: 610 nm) of PI is shown on the ordinate. Viable cells, with a high mitochondrial membrane potential, are located in the lower right quadrant (Q4) of the dot plots. Cells showing dissipation of the ΔΨm potential and an intact plasma membrane can be identified in the lower left quadrant (Q3). Degenerating cells, with depolarized mitochondria and deteriorated plasma membrane, are located in the upper left quadrant (Q1) of the dot plots. The numerical values shown within the quadrants refer to the mean percentage of cells in the respective conditions, which was calculated on the basis of three independent determinations.

#### 2.7.2. Morphological Evaluation of Mitochondria

For this analysis, two main types of mitochondrial morphology, rounded/fragmented and elongated, were evaluated. Counting was performed on fluorescence images, and at least 200 cells from different microscopic fields, obtained from three slides for each experimental condition, were evaluated. Using SigmaScan^®^ Pro 5.0 image analysis software (Systat Software, Inc., San Jose, CA, USA), two parameters were derived and represented in their respective graphs. The first parameter refers to the percentage quantification, in the total population examined, of the fraction of cells in which rounded/fragmented mitochondria predominate. In the case of the second parameter, the quantification provides an indication of the percentage variation in the ratio between mitochondria with rounded and elongated morphology within the cytoplasm of the single cell.

### 2.8. Superoxide Anion Detection at the Mitochondria Level: Cell Imaging and Microfluorometric Measurements

Mitochondrial superoxide anion production in viable cells was visualized by fluorescence microscopy and measured by microfluorometry using MitoSOX Red (Invitrogen, Eugene, CA, USA). This probe consists of the lipophilic cation triphenylphosphonium (TPP+) bound to a hydroethidine (DHE) moiety. Due to the negative potential of the inner mitochondrial membrane (ΔΨm), TPP+ preferentially accumulates inside the mitochondria, so that DHE can form a specific, fluorescent product (after binding to mitochondrial DNA) by reaction with superoxide radical anions. In general, the high concentration of MitoSOX in the mitochondrial matrix also allows the dye to compete with the enzyme MnSOD (Manganese SuperOxide Dismutase), an endogenous mitochondrial scavenger [21].

To determine the levels of mitochondrial superoxide anion in the cultures under different experimental conditions, the MitoSOX Red probe was directly added to the culture medium at a concentration of 2 µM. The cells that adhered to the glass coverslips were subjected to microfluorometric measurements or image acquisition after an incubation time of 20 min at 37 °C. The living cells were imaged using a Nikon Eclipse 600 microscope (Nikon, Kanagawa, Japan), equipped with cubes with interchangeable filters for different fluorescence. To obtain an immediate visualization of the presence of MitoSOX-negative cells, the nuclei were counterstained with Hoechst 33342. Images were then obtained by applying the following spectral conditions: the red fluorescence of MitoSOX was excited at 510 nm with emission at 610 nm, whereas the blue fluorescence of Hoechst 33342 (Sigma-Aldrich, Saint-Louis, MO, USA) was excited at 346 nm with emission at 460 nm. Each image was acquired at 50× magnification using a camera (Q-imaging, Aqua Exi, 14 bit, 6.45 μm per pixel, Adept Turnkey, Perth, Australia) with an appropriate exposure time set to avoid saturation and bleaching.

Fluorescence measurements of cells labeled with MitoSOX Red were carried out with a Nikon P II microfluorometer (Nikon, Kanagawa, Japan) under the same spectral conditions used for fluorescence microscopy. In both untreated and hydrogen peroxide-treated cells, preliminary tests allowed us to establish that, under appropriate illumination calibration conditions, the fluorescence of the MitoSOX Red probe remained constant within approximately 70 min after the end of labeling. For the microfluorometric measurements, a 100× oil immersion objective was used. An appropriate diaphragm system allowed the selection of individual cells. Fluorescence was analyzed quantitatively after automatic subtraction of background brightness. The operations of alignment and focusing of the cells were conducted preliminarily in phase contrast microscopy, under low light conditions, to avoid photo-decay. For each experimental condition, the mean fluorescence intensity was obtained by measuring 200 cells on three different slides, after subtraction of background signals and correction for the brightness of unlabeled cells. The fluorescence measurements of MitoSOX Red are represented as frequency distribution curves of intensities obtained from the respective histograms and expressed numerically as mean values for any cell fraction present. The threshold below which the fluorescence intensity values fall between those detected in the background and in the cells negative for labeling is indicated in the graphs.

### 2.9. Morphological and Functional Aspects Related to the Lysosomal Compartment

#### 2.9.1. Microfluorometric Measurements of Lysosome Content/Activity and Lipofuscin Autofluorescence

Lysosomes and acidic vesicles related to these organelles were visualized in viable cells using the low molecular weight fluorescent probe Lysotracker Red (LTR) (Invitrogen, Eugene, CA, USA). Alternatively, we used the metachromatic fluorochrome Acridine Orange (AO) (Sigma-Aldrich, Saint-Louis, MO, USA), which, under appropriate conditions, is also able to provide information on the involvement of the lysosomal compartment in autophagic events. Lysotracker Red passes easily through plasma membranes and cytoplasmic vesicle membranes and is retained within them due to low pH. Here, the fluorophore, bound to a weak base, is protonated, fluoresces, and is unable to cross the vesicle membranes again. For the fluorochromization of Lysotracker Red, cells derived from the different experimental conditions were grown on coverslips placed on the bottom of multi-well plates. The cells were incubated for 30 min. at 37 °C with a 50 nM probe solution in culture medium. For microfluorometric measurements and microscopy, Lysotracker Red fluorescence was excited at 570 nm and collected at 590 nm. The amount of lysosomes per cell was analyzed by measuring the red fluorescence emitted by every individual cell. A Nikon P II microfluorometer (Nikon, Kanagawa, Japan) with a 100× oil immersion objective was used for this analysis. In the microfluorometric equipment, an appropriate diaphragm system allowed individual cells to be selected, and the fluorescence was analyzed quantitatively after automatic subtraction of the background brightness. The operations of alignment and focusing of the cells were preliminarily conducted in phase contrast, under low light conditions, to avoid photo-decay. The yellow-green autofluorescence of lipofuscins (LPFs) was detected by exciting the same cells at 450–490 nm, using a dichromatic mirror at 510 nm and a long-pass filter at 515 nm [22].

For each experimental condition, three slides were analyzed, and 200 cells were measured in each. Lysosomal fluorescence and lipofuscin autofluorescence measurements are represented as frequency distribution curves of intensities obtained from the respective histograms and are expressed numerically as mean values for any cell fraction present. In the case of the lipofuscin autofluorescence measurements, in the graphs the threshold below which the intensity values fall between those detected in the background and in non-autofluorescent cells is shown. In our experience, we found no significant differences in the overall trend of the curves obtained, when the two fluorescence measurements were performed on parallel samples and not on the same cells. This excluded any photo-decay due to the excitation of the two fluorescence in the same cell. Furthermore, no significant difference was found by first measuring one or the other of the two types of fluorescence in the same cell.

#### 2.9.2. Morphology and Co-Localization of Lysosomal Compartment Fluorescence and Lipofuscin Autofluorescence

By means of Lysotracker Red (LTR) fluorescence and cytoplasmic autofluorescence of lipofuscin (LPF) inclusions, it was possible to obtain, in the same cell, separate microscopy images of the two types of light emissions. In addition to the respective morphological aspects, it was possible to obtain a quantitative estimate of large areas (aggregates) of the labeling.

For the measurements, the two fluorescent cytoplasmic areas and the boundaries of the plasma membrane (total cell area) were manually marked using ImageJ analysis software 1.54p (ImageJ: Rasband, W.S., ImageJ, U. S. National Institutes of Health, Bethesda, MD, USA. https://imagej.net/ij/, 1997–2018) (accessed on 17 March 2025).

The measured values of the lysosomal fluorescence aggregation index (LYAI) and the lipofuscin autofluorescence aggregation index (LPAI) were normalized by comparing them to the total area of the individual cells analyzed and are expressed as percentages.

To evaluate the co-localization of lipofuscin accumulation (green fluorescence) with the lysosomes (red fluorescence), merged images of the two types of fluorescence, in the respective experimental conditions, were examined. The overlap of the two fluorescence signals results in a yellow color and appears in the form of dots or aggregates. The values of the co-localization index of the two fluorescence signals (COLI) obtained from the measurement of the overlapping yellow areas are recorded. The measurement of yellow spots per cell was normalized taking into account the total cell area and expressed as a percentage relative to the red fluorescence of the lysosomal compartment.

For these analyses, at least 200 cells per microscopic field were measured.

#### 2.9.3. Detection of Senescence-Associated SA-β-Galactosidase Activity

Depending on its localization in lysosomes, β-galactosidase shows its maximum activity at pH values between 4.0 and 4.5. The enzyme activity is significantly lower at pH 6.0 and is usually not detectable in actively proliferating cells by in situ staining at this pH value. The detection of Senescence-associated-β-galactosidase (SA-β-gal) activity at pH 6.0 is a commonly used biomarker method for the identification and characterization of senescent cells. In this work, the enzymatic activity of SA-β-gal was assessed by means of a chromogenic reaction. For this procedure, cells derived from the different experimental conditions—grown on glass coverslips, after being washed in PBS (Sigma-Aldrich, Saint-Louis, MO, USA) to remove traces of culture medium—were fixed with 3% paraformaldehyde (Sigma-Aldrich, Saint-Louis, MO, USA) for 5 min at room temperature. After washing to remove traces of fixative, the enzymatic activity of SA-β-galactosidase was detected using the chromogenic substrate 5-bromo-4-chloro-3-indolyl-β-D-galactopyranoside (X-gal) (Invitrogen, Eugene, CA, USA), at concentration of 1 mg/mL, in a solution containing 40 mM citric acid/sodium phosphate (pH 6) (Sigma-Aldrich, Saint-Louis, MO, USA), 5 mM potassium ferricyanide (Sigma-Aldrich, Saint-Louis, MO, USA), 150 mM NaCl (Sigma-Aldrich, Saint-Louis, MO, USA), and 2 mM MgCl_2_. (Sigma-Aldrich, Saint-Louis, MO, USA). Blueish-green cytoplasmic staining was already evident after approximately 3 h and increased in intensity in the following hours. The micrographs were obtained in phase contrast, at 200–400× magnification, after adding a drop of mounting medium (Dako, Carpinteria, CA, USA) to the slide. For each experimental condition, three different slides were evaluated. Samples with at least 80% positive cells for SA-β-gal were generally considered senescent. The percentage of cells positive for the chromogenic reaction was evaluated after at least 200 cells per microscopic field were measured. The positivity threshold was determined after evaluating the occasional weak staining in some cells of the untreated NIHb culture. To determine the threshold values, the bluish/green cytoplasmic areas were compared to the area occupied by the entire cell. For measurements, the stained cytoplasmic areas and the boundaries of the plasma membrane (total cell area) were manually marked using ImageJ analysis software 1.54p (accessed on 30 June 2025). In the various experimental conditions, all cells with a greater bluish-green area and more intense staining, compared to those detected in untreated NIHb cells, were considered positive. These cells were represented, in the graphs, as percentage fractions in comparison with the totality of the cell populations examined. The changes in bluish-green staining were also evaluated in single cells in terms of both the overall positive cytoplasmic area and the intensity of labeling. The values detected in the individual cells were normalized considering the respective total cell areas.

#### 2.9.4. Autophagy Detection

An estimation of autophagic events can be obtained using Acridine Orange fluorochrome (Sigma-Aldrich, Saint-Louis, MO, USA) under cell viability conditions. Through a specific incubation procedure (low concentration and neutral pH), AO accumulates in the acidic compartments of the cells as a function of pH. Inside acidic vesicles, such as lysosomes and autolysosomes (pH values between 4.5 and 5.0), the molecule is protonated and forms dimers that emit bright red fluorescence. Under these incubation conditions, the permeability of the membranes to the protonated AO is very low; thus, it is possible to clearly distinguish the fluorescent red granulations in the cytoplasm. This method is commonly used not only to visualize acidic intracellular compartments, but also to quantify their volume as a result of increased events such as autophagy [23]. This assay can be an alternative or complement to other methods for determining acidic vesicles during the induction of autophagy. Other work has indicated that fluorescent red granulations in the cytoplasm, obtained after AO staining under the conditions described above, are correlated with the distribution of fluorescent-tagged LC3 [23]. In addition, compared with other lysosome-specific probes, Acridine Orange exhibits metachromatic properties with the emission of a second weak green fluorescence that marks not only the nucleus but also the entire cytoplasm, thus allowing information on cell morphology to be obtained. For staining procedure used in this work, the cells were incubated for 10 min at 37 °C with AO (2.5 μg/mL in culture medium). The samples were examined after washing in PBS to remove excess dye. At least 200 cells from three different slides were evaluated for each experimental condition. The analysis was performed under a fluorescence microscope with a 50× oil immersion objective. The percentage of cells involved in autophagic events was determined by considering the positivity threshold of a parameter called Mean Autophagic Index (MAI). To derive this parameter, the cytoplasmic red fluorescence produced by AO fluorochromization was taken into consideration. As part of this labeling, emphasis was given exclusively to the presence of red cytoplasmic areas of larger dimensions compared to the punctiform labeling of the lysosomes. These areas were considered indicative of the presence of autophagic events. The values measured per single cell were normalized by comparing them with the overall area occupied by the cell. The latter was measured after the detection of the cell boundaries indicated by the weaker cytoplasmic green fluorescence. The different measurements were performed using SigmaScan^®^ Pro 5.0 image analysis software (Systat Software, Inc., San Jose, CA, USA). The positivity threshold of 2.30 was established by taking into account the MAI value measured most frequently in the cells of the NIHb culture in the absence of hydrogen peroxide treatment. In this way, all cells from different cultures in which an MAI value greater than 2.30 was detected were considered autophagic, and their number was expressed as a percentage of the total cells examined.

### 2.10. Statistical Analysis

Statistical analysis was performed using SigmaPlot Software (Systat Software Inc., San Jose, CA, USA). The measurement data are expressed as mean ± standard error (SEM). For the comparison between two groups, Student’s *t*-test was used. *p* < 0.05 was considered statistically significant.

## 3. Results

### 3.1. Morphology and Adhesive Properties of Different Cultures During Selection, Enrichment, and Propagation Phases

**Figure 1:**
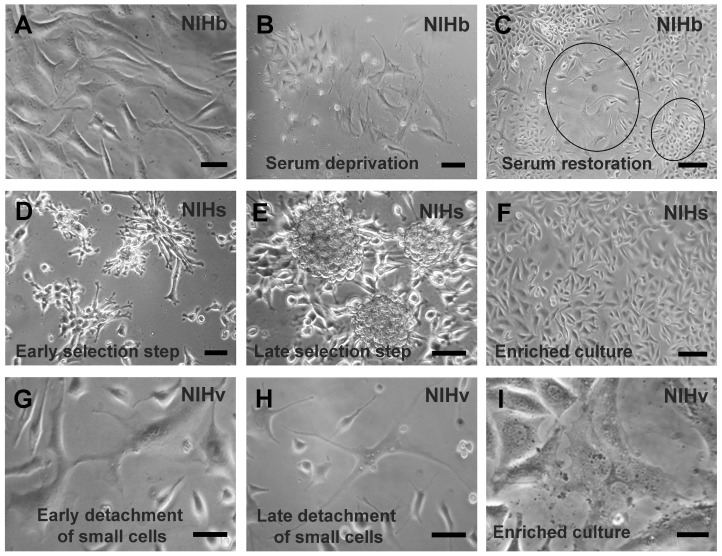
*The morphology of the NIHb, NIHs, and NIHv cultures. The selection and enrichment of NIHs and NIHv cells.* The phase contrast micrographs show the cultures during the selection and enrichment phases of the different cell types and the typical morphology of the NIHb, NIHs, and NIHv cells used in this work. In (**A**), the original NIH/3T3 line, here called NIHb, is visible. In (**B**), the morphology of cells remaining in the culture after the massive cell detachment induced by serum starvation is shown. Cells with different characteristics can be identified. In (**C**), the image shows the culture after the resumption of the proliferative activity in the presence of the serum; the prevailing type is represented by smaller, highly proliferating cells. In some areas of the growth substrate, flattened cells with a larger cytoplasm (ellipsoidal areas) were also identified. From these two cell types, NIHs and NIHv cultures were derived, respectively. In (**D**,**E**), the phases of the culture propagation during the selection of NIHs cells are shown (see Section 2). In particular, in (**D**) the foci of actively proliferating cells can be observed in some areas of the growth substrate, whereas in (**E**) spheroids originating from the detachment and proliferation of cells from these areas are visible. In (**G**,**H**), the stages of culture propagation during the selection of NIHv cells are shown (see Section 2): in particular, the subsequent stages of the detachment of the smaller cells after trypsinization can be observed. In this way, the NIHv culture was enriched with more adherent cells with an extended cytoplasm. The morphology of the NIHs and NIHv cultures is shown in (**F**,**I**). Scale bars in (**A**,**C**,**I**): 10 μm; in (**B**,**D**–**H**): 20 μm.

The morphological results shown below are derived from observations made under a phase contrast microscope (Figure 1).

As described in Section 2, NIHs and NIHv cultures were obtained from the original NIH/3T3 line, here called NIHb. The cells of this line exhibited a classical fusate and stellate morphology (Figure 1A). The serum deprivation of the NIHb culture, prolonged for 7–10 days, induced massive cell detachment. Among the cells that remained adherent to the growth substrate, two main types were observable: the first was homogeneous in shape and smaller in size, and the second was larger in size and had a more variable morphology (Figure 1B). When the serum was restored to the culture medium, the two cell types presented different behaviors.

The smaller cells readily recovered their proliferative activity and became the predominant fraction of the culture (Figure 1C). The larger cells showed a slow proliferation and a flattened morphology with extended cytoplasm. These cells presented a greater adhesion capacity than the prevailing smaller cells and were identifiable only in certain areas of the growth substrate (Figure 1C). During the enrichment and propagation phases of the cultures (see Section 2), the smaller cells were more easily detached by trypsinization procedures, whereas the larger cells were more difficult to detach from the growth substrate. From these cell types, two cultures were derived, named NIHs and NIHv, respectively, in which the different adhesive capacities were inversely correlated with the proliferative activity. In the micrographs in Figure 1D,E, the stages of the culture propagation during the selection of NIHs cells are shown. In particular, the tendency to form foci of actively proliferating cells in certain areas of the growth substrate can be observed (Figure 1D). In addition, spheroids originating from cells detached and proliferating from these areas were visible (Figure 1E). After isolation, the spheroids were dissociated, and NIHs cell monolayers were obtained (Figure 1F). The micrographs in Figure 1G,H, on the other hand, showed several steps in the selection of NIHv cells, where their high adhesive capacity was exploited in their enrichment procedure. In particular, the subsequent stages of the detachment after the trypsinization of more proliferating, smaller, and less adherent cells can be observed (Figure 1G,H). After the removal of the smallest and least adherent cells by aspiration, the NIHv culture was enriched with more adherent cells (Figure 1G,H). The predominant morphology of the NIHv culture obtained was characterized by an extensive and flattened cytoplasm (Figure 1I). The prolonged trypsinization times required for their detachment were consistent with the morpho-functional properties described above (for details, see Section 2).

### 3.2. The Comparison of the Proliferative Activity and Cell Cycle Progression of NIHb, NIHs, and NIHv Cultures

**Figure 2:**
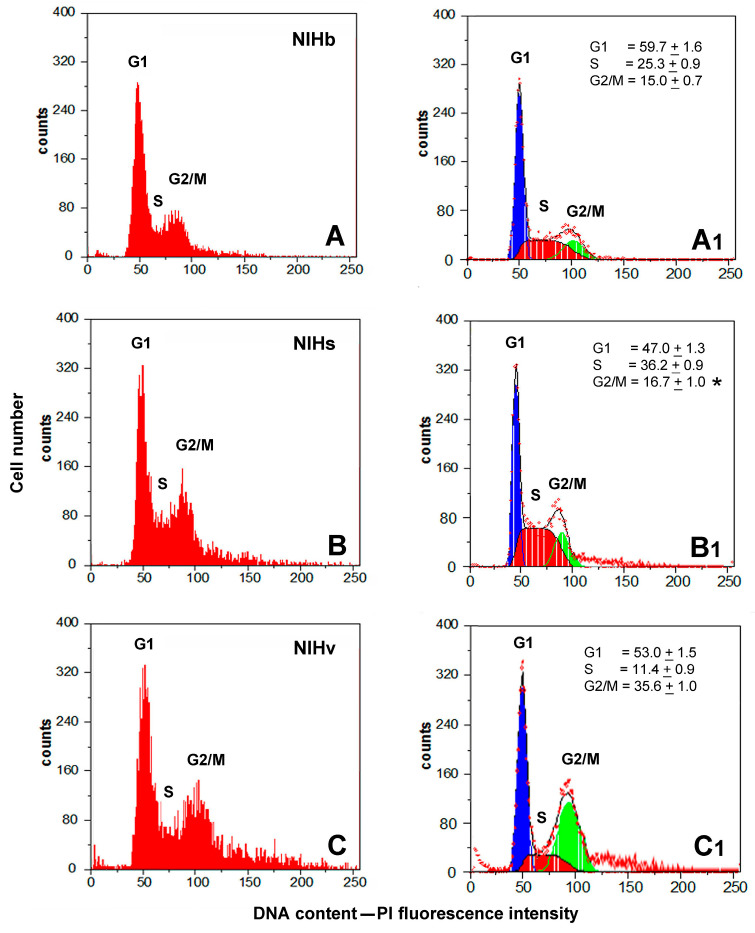
The cytofluorimetry of the DNA content in NIHb, NIHs, and NIHv cultures. DNA content measurements were performed on cells from NIHb (**A**), NIHs (**B**), and NIHv (**C**) cultures after fluorochromization with propidium iodide (PI). The percentage of cells in distinct phases of the cell cycle, shown in (**A1**–**C1**), was calculated after the deconvolution of the DNA content frequency histograms, shown in (**A**), (**B**), and (**C**), respectively. In A1–C1 the blue, red and green areas represent the percentage of cells that are in the G1, S and G2/M phases of the cell cycle, respectively. In the histograms, the fluorescence intensity of propidium iodide is shown on the X-axis, on a linear scale. The numerical data shown in the graphs refer to the percentage of cells in the G1, S, and G2/M phases and are the means (±SE) of the values obtained from three independent experiments (*n* = 3). In the comparison between the different cultures (NIHs vs. NIHb cells and NIHv vs. NIHb cells), the means were significantly different (*p* < 0.05 after Student’s *t*-test) except for the values marked with an asterisk (*).

**Figure 3:**
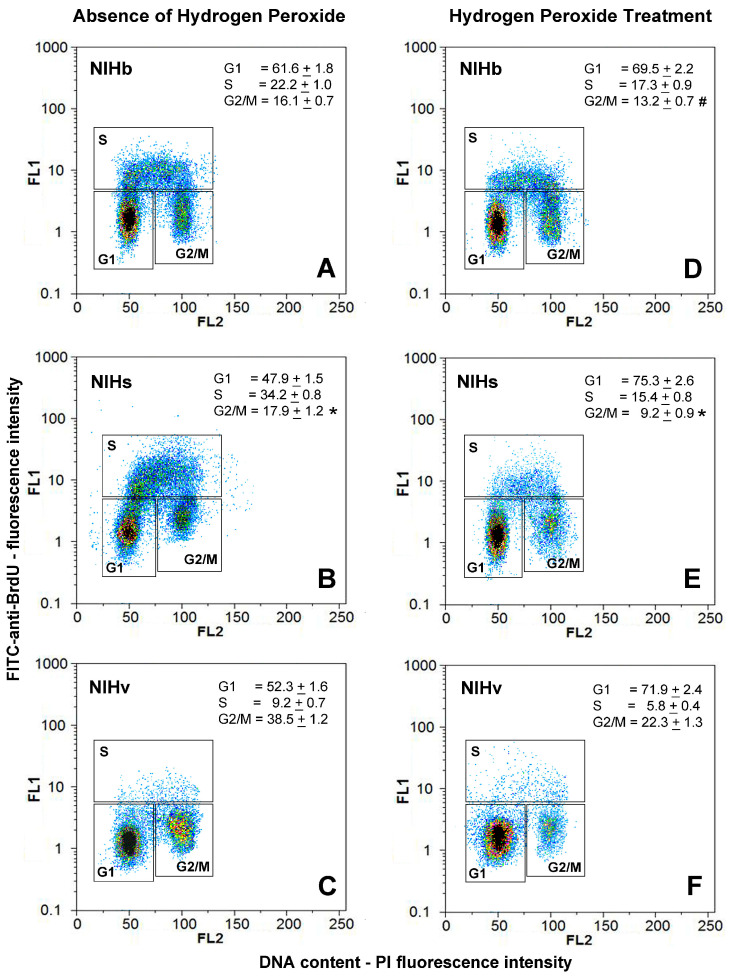
*The cytofluorimetry of the BrdU uptake in cultures of NIHb, NIHs, and NIHv, before and after the treatment with hydrogen peroxide*. A series of representative dot plots of the cell cycle analysis after the fluorochromization with propidium iodide and FITC-anti-BrdU immunoreaction are shown. Dot plots (**A**,**D**) refer to NIHb cells, (**B**,**E**) refer to NIHs cells, and (**C**,**F**) refer to NIHv cells. Flow cytometric measurements were performed before (**A**–**C**) and after (**D**–**F**) the hydrogen peroxide treatment. The fluorescence intensity of propidium iodide is reported on the X-axis (FL2), on a linear scale, whereas the fluorescence intensity of FITC-anti-BrdU is displayed, on a logarithmic scale, on the Y-axis (FL1). The selection of the areas of the PI/FITC-anti-BrdU spots by means of a gating process made it possible to establish the percentage distribution of the cells in the phases of the cell cycle (G1, S, and G2/M). The numerical data shown in the graphs refer to the percentage of cells in the G1, S, and G2/M phases and are the means (±SE) of the values obtained from three independent experiments (*n* = 3). In the comparison between the different cultures (NIHs vs NIHb cells and NIHv vs NIHb cells), both before and after treatment with hydrogen peroxide, the means were significantly different (*p* < 0.05 after Student’s *t*-test) except for the values marked with an asterisk (*). By comparing the same culture type, before and after treatment with hydrogen peroxide (e.g. +H_2_O_2_ NIHb vs −H_2_O_2_ NIHb), the means were significantly different (*p* < 0.05 after Student’s *t*-test) except for the values marked with hashtag (#).

To obtain preliminary information on the proliferative activity of the cells constituting the NIHb, NIHs, and NIHv cultures, the DNA content was analyzed by flow cytometry after fluorochromization with propidium iodide. The flow cytometry data were reported as frequency histograms of the DNA content (PI-fluorescence intensity) of the different cultures (Figure 2A–C). The percentage of cells in distinct phases of the cell cycle (Figure 2(A1–C1)) was calculated after the deconvolution of the DNA content histograms using FlowMax software. This analysis provides a static image of the percentage of cells in the different phases of the cell cycle. The red areas subtended by the frequency curves (Figure 2(A1–C1)), which represent the percentage of cells in the S phase, do not provide information on the fraction of nonproliferating elements that may be blocked in this phase of the cell cycle. Therefore, to obtain more detailed information on the percentage of truly proliferating cells, PI fluorochromization was subsequently combined with FITC-conjugated anti-BrdU antibody staining (Figure 3A–C).

Before the immunoreaction, the cells were incubated with the thymidine analog BrdU, which is incorporated into DNA during its replication (see Section 2). Flow cytometric data of the double fluorochromization were represented in two-dimensional dot plots showing the cells stained with propidium iodide and FITC-conjugated anti-BrdU antibodies (Figure 3). The fluorescence intensity of PI is plotted on a linear scale on the X-axis, whereas the fluorescence intensity of FITC-BrdU is measured on a logarithmic scale on the Y-axis. The selection of PI/ FITC-BrdU dot plot areas by the gating process allowed us to establish the percentage distribution of cells in the cell cycle phases (G1, S, and G2/M). Despite the possible limitation produced by the monoparametric evaluation of the DNA content, the percentage of cells in the S phase, calculated after the BrdU incorporation, was only slightly lower, with a good correlation (r = 0.95) between the values measured with the two methodologies.

The flow cytometric analysis of the cell cycle in monoparametric (Figure 2(A–C,A1–C1)) and biparametric (Figure 3A–C) modes revealed differences in the progression of the cytokinetic activity in the NIHs and NIHv cultures, compared with the reference NIHb cultures, with variations in the percentage of cells in the different phases of the cell cycle. The greater proliferative activity of the NIHs cells, compared with other cultures, was demonstrated by the higher percentage of cells in the S phase of the cell cycle (Figure 2(B1) and Figure 3B). Compared with those of the other cultures, flow cytometric measurements of the NIHv culture revealed the lowest percentage of cells in the S phase (Figure 2(C1) and Figure 3C). The decrease in the percentage of cells in the S phase was not accompanied by a reduction but rather by an increase in the percentage of cells in the G2/M phase (Figure 2(C1) and Figure 3C). Compared with the NIHb culture (Figure 2(A1) and Figure 3A), for both the NIHs (Figure 2(B1) and Figure 3B) and NIHv (Figure 2(C1) and Figure 3C) cultures, the change in the percentage of cells in the S phase resulted in a significant decrease in the number of cells in the G1 phase of the cell cycle.

### 3.3. PI and BrdU Staining and Cell Cycle Analysis Before and After Hydrogen Peroxide Treatment

The combined PI and FITC-BrdU fluorochromization was used to evaluate the effects induced by the hydrogen peroxide treatment in the different cultures. The analysis of the cell cycle after the incubation with the oxidizing agent (Figure 3D–F) revealed effects that were at least partially dependent on the cytokinetic activity under basal conditions (Figure 3A–C). An accumulation of cells in the G1 phase of the cell cycle was observed in all cultures (Figure 3D–F). The effect, which differed in magnitude across the three cultures, could be due to the lack of a transition between the G1 and S phases of the cell cycle. This would cause a variable decrease in the percentage fraction of cells that reach the S phase (Figure 3D–F) compared with the untreated condition (Figure 3A–C). A comparison between the NIHb reference culture and the transformed NIHs culture revealed that, under basal conditions, the percentages of cells in the S phase were 22.2% and 34.2%, on average, respectively (Figure 3A,B). In both the NIHb and NIHs cultures, the hydrogen peroxide treatment induced a decrease in the percentage of cells in the S phase of the cell cycle, with mean values of 17.3% and 15.4%, respectively (Figure 3D,E). Despite the closeness of these latter percentage values, the different significance of the effect should be emphasized. Comparing only the S phase cell cohorts, a reduction of approximately 22% was detected in the NIHb culture, whereas the value is approximately 45% in the NIHs culture. With respect to the senescent NIHv culture, the analysis of the entire cell cycle indicated that 9.2% of the cells, on average, were detected in the S phase, in the absence of the treatment (Figure 3C), whereas after the incubation with hydrogen peroxide, the value was 5.8%, on average (Figure 3F). The percentage of NIHv cells that reached the G2/M phase was 38.5% on average in the absence of the treatment (Figure 3C) and 22.3%, on average, after the incubation with hydrogen peroxide (Figure 3F). This decrease could depend on the even more limited number of cells capable of entering the S phase of the cell cycle. Even the occurrence of mitotic catastrophe events, in those cells that managed to complete the DNA duplication, could contribute to this decrease (for morphology see below).

In summary, hydrogen peroxide induced primary effects on G1 phases resulting in arrested proliferative activity. However, the extent of these effects seemed to be dependent on the specific basal cytokinetic characteristics of the different cultures, with the possible involvement of other phases of the cell cycle. In particular, in NIHv cells, the G2/M phase was also affected. The cytometric data of the cell accumulation in this phase of the cell cycle are supported by the detection of the multinucleation and mitotic catastrophe. Mitotic alterations of this type, as well as multinuclear conditions, were detected in NIHv cells even before the treatment with hydrogen peroxide (for morphology see below).

Rare conventional apoptotic death events were detected under all experimental conditions, without influencing the different cytokinetic dynamics.

### 3.4. Content/Functionality and Morphology of Mitochondria Before and After Treatment with Hydrogen Peroxide

**Figure 4:**
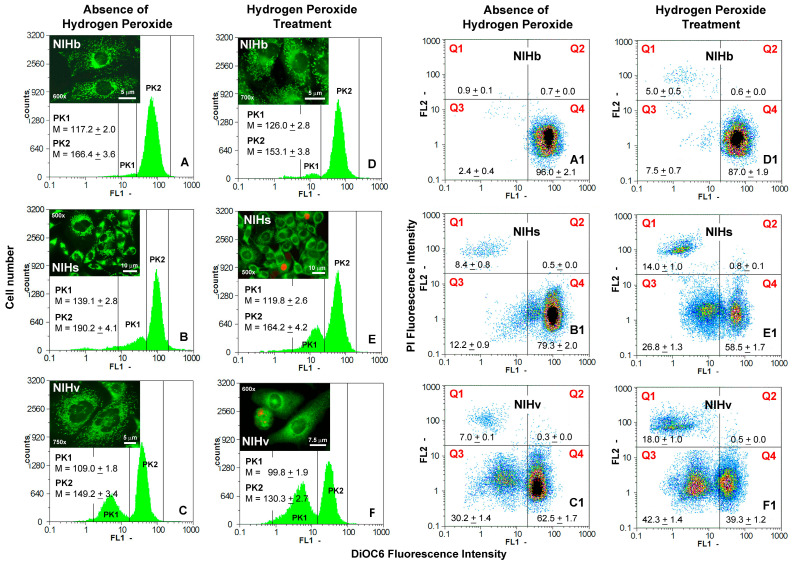
*The cytofluorimetry and morphology of the mitochondria, before and after the treatment with hydrogen peroxide.* The correlation with the plasma membrane functionality. The histograms show the DiOC6 fluorescence of the mitochondria before (**A**–**C**) and after (**D**–**F**) the hydrogen peroxide treatment, in NIHb (**A**,**D**), NIHs (**B**,**E**), and NIHv (**C**,**F**) cells. In the dot plots, the fluorescence of DiOC6 (FL1) and PI (FL2), referred to as NIHb, NIHs, and NIHv cells before (**A1**–**C1**) and after (**D1**–**F1**) the treatment, allowed us to correlate the mitochondrial content/activity with the plasma membrane functionality. In (**A**–**F**), numerical values indicate the mean DiOC6 fluorescence intensity (M) of unimodal and/or multimodal distributions expressing cell fractions (PK1 and PK2) with a different mitochondrial content/activity. All numerical values represent the means ± SE of triplicate determinations (*n* = 3). In the comparison between untreated cultures, NIHs (**B**) vs. NIHb (**A**) and NIHv (**C**) vs. NIHb (**A**), as well as, for the same culture, between treated and untreated cells (NIHb: (**D**) vs. (**A**); NIHs: (**E**) vs. (**B**); and NIHv: (**F**) vs. (**C**)), the means were significantly different (*p* < 0.05 after Student’s *t*-test). In the dot plots, the Q4 quadrant represents viable cells (high ΔΨm), the Q3 quadrant represents cells with ΔΨm dissipation and an intact plasma membrane, and the Q1 quadrant represents degenerating cells, with depolarized mitochondria and deteriorated plasma membrane. Comparing the quadrants of untreated NIHs cells and NIHv and NIHb cells ((**B1**) vs. (**A1**); (**C1**) vs. (**A1**)), as well as before and after the treatment of the same culture type ((**D1**) vs. (**A1**); (**E1**) vs. (**B1**); (**F1**) vs. (**C1**)), the means were significantly different (*p* < 0.05 after Student’s *t*-test). In the insets, the green fluorescence shows the morphology/distribution of the mitochondria before (**A**–**C**) and after the treatment (**D**–**F**) in NIHb (**A**,**D**), NIHs (**B**,**E**), and NIHv (**C**,**F**) cells. In (**E**,**F**), the red labeling of the nuclei indicates the PI uptake due to alterations in the plasma membrane. Scale bars and image magnification: (**A**), 5 μm (600×); (**B**), 10 μm (500×); (**C**), 5 μm (750×); (**D**), 5 μm (700×); (**E**), 10 μm (500×); and (**F**), 7.5 μm (600×).

The analysis of the mitochondrial compartment was carried out under cell viability conditions using the fluorescent cationic probe DiOC6, which is quantitatively retained in the mitochondrial matrix as a function of the ΔΨm potential established during the respiratory activity. The fluorescence measurement by flow cytometry provides information on the mitochondrial content/activity, a useful indicator for dynamically assessing the functional state of cells cultured under different experimental conditions. The fluorochromization with DiOC6 was performed in the presence of propidium iodide. The simultaneous analysis of both fluorescence signals allowed us to identify, in the dot plots, cell fractions in which the mitochondrial content/activity could be correlated with the level of the PI-permeability of the plasma membrane and thus with its functional status (Figure 4).

The quantitative cytofluorimetric data were integrated with the morphological information obtained by the fluorescence microscopy. Flow cytometric measurements of the mitochondrial fluorochromization performed on NIHb, NIHs, and NIHv cultures, before (Figure 4A–C) and after (Figure 4D–F) the hydrogen peroxide treatment, are shown in frequency histograms of the fluorescence intensity of DiOC6. A comparison of these graphs allows us to highlight some differences in the frequency distributions and values of the mean fluorescence intensity. Under certain experimental conditions, a bimodal pattern, more or less accentuated, was found, indicating the presence of cell fractions with a different mitochondrial content/activity (lower for PK1 and higher for PK2 peaks) (Figure 4). The bivariate analysis of the flow cytometric data after the fluorochromization of DiOC6/PI is represented as dot plots of the two fluorescence signals and provides information on the percentage incidence of the different cell fractions before (Figure 4(A1–C1)) and after (Figure 4(D1–F1)) the hydrogen peroxide incubation. The PK1 and PK2 peaks present in the histograms shown in Figure 4A–F largely represent cells that are positioned in the Q3 and Q4 dot plot quadrants, respectively. The fractions included in these two quadrants, although exhibiting different intensities of mitochondrial fluorescence (lowest in Q3; highest in Q4), are both made up of cells that do not incorporate propidium iodide.

In the NIHb reference culture, in the absence of treatment, a mean fluorescence intensity value of 166.4 (PK2), Figure 4A, was recorded for 96.0% of the cells on average (Figure 4(A1)). This set of cells excluding propidium iodide can be identified in the Q4 quadrant at the bottom right of the corresponding dot plot (Figure 4(A1)).

The treatment with hydrogen peroxide induced a limited effect on the mitochondrial content/activity (Figure 4D): NIHb cells showed a reduction in fluorescence intensity with a mean value of 153.1 (PK2) (Figure 4D). Compared with that of the untreated cells, the DiOC6 signal decreases by approximately 8%. The examination of the dot plot of the treated NIHb culture (Figure 4(D1)) revealed that approximately 12% of the NIHb cells (sum of the percentage values in quadrants Q1 and Q3) showed a lower mitochondrial fluorescence intensity value. The latter detected in the histogram of Figure 4D is equal to 126.0, on average (PK1). In the Q1 quadrant, at the top left of the dot plot in Figure 4(D1), PI-uptaking cells can be identified, with a mean incidence of 5.0%. A further evaluation can be derived from the analysis of the cohort made up of the cells present in the Q4 and Q1 quadrants. The fractions in Q4 and Q1 represent the cells that show the highest or lowest ratio between the mitochondrial content/activity and PI uptake, respectively. A comparison of the conditions before and after the hydrogen peroxide treatment indicated that, in NIHb cells, the shift in the Q4/Q1 proportions increases from approximately 0.9 to approximately 5.4 (Figure 4(A1,D1)).

Compared with the other cultures, in the NIHs cultures, in the absence of treatment, the predominant fraction of cells showed the highest mean fluorescence intensity value of 190.2 (PK2) (Figure 4B). In the corresponding dot plot, the percentage shown in quadrant Q4 (79.3% on average) included cells with these characteristics but also a detectable sub-fraction with a lower fluorescence intensity (Figure 4(B1)). In the histogram in Figure 4B, these latter cells are reasonably represented by the signals within the PK1 peak, which expresses a mean mitochondrial fluorescence value of 139.1. In quadrant Q3 of Figure 4(B1), with a mean incidence of 12.2%, the cells with the lowest mitochondrial fluorescence can also be identified. Except for a fraction of the cells (8.4% on average), located in the upper left quadrant Q1 (Figure 4(B1)), all the NIHs cells were negative for the PI uptake.

The treatment with hydrogen peroxide caused a reduction in the mitochondrial fluorescence in the NIHs culture. The mean intensity value, measured in the histogram, was 164.2 (PK2) (Figure 4E). Compared with that of the untreated NIHs cells, the fluorescence signal of DiOC6 decreased by approximately 14%. The corresponding dot plot (Figure 4(E1)) shows that 58.5%, on average, of the cells in this situation (Q4 quadrant) are PI-negative. Compared with the NIHb culture, the incubation with the oxidizing agent resulted in a more pronounced increase in the fraction of cells with low mitochondrial fluorescence, with a mean intensity value of 119.8 (PK1) (Figure 4E). These NIHs cells, which are also PI-negative, can be identified in the Q3 quadrant of the respective dot plot, with an incidence of 26.8% (Figure 4(E1)). The only cells (14.0% on average) with an altered plasma membrane and very low mitochondrial fluorescence after treatment were identified in quadrant Q1. A comparison of the conditions before and after the hydrogen peroxide treatment indicated that the shift in the Q4/Q1 proportions increased from approximately 9.6 to approximately 19.3 (Figure 4(B1,E1)).

Also, in the case of the NIHv culture, in the absence of treatment, cytometric measurements of the DiOC6 fluorescence clearly revealed a bimodal histogram pattern, indicating the presence of cell fractions with different mitochondrial content/activities (PK1 and PK2 peaks) (Figure 4C). The predominant one showed a mean fluorescence intensity value of 149.2 (PK2), whereas in the other fraction the value was 109.0 (PK1) (Figure 4C). These two PI-negative cell fractions can also be identified (with mean incidences of 62.5 and 30.2%) in the lower part of the corresponding dot plot (Figure 4(C1)), in the right (Q4) and left (Q3) quadrants, respectively. The percentage of PI-positive NIHv cells, shown in the upper left quadrant Q1 of the dot plot, was 7.0% on average (Figure 4(C1)).

The hydrogen peroxide treatment of the NIHv culture resulted in a general decrease in the mitochondrial content/activity in all cells, with a significant increase in the cell fraction with a low mitochondrial content/activity (Figure 4F). In contrast to the findings in the NIHs culture (Figure 4(E,E1)), in the NIHv culture the two main cell fractions, with a low (M = 99.8) and high (M = 130.3) mitochondrial content/activity (Figure 4F), tended to balance each other in the histograms. In the prevalent cellular fraction, the fluorescence signal of DiOC6 compared to untreated NIHv cells decreased by approximately 13%.

Their presence can also be deduced in the corresponding dot plot (Figure 4(F1)) in quadrants Q3 and Q4, with mean incidences of 42.3% and 39.3%, respectively. In the NIHv culture, the incubation with hydrogen peroxide induced the presence of the largest fraction of cells with an altered plasma membrane (high level of PI uptake), showing a threefold increase in the mean value (18.0%) (Q1 quadrant in the upper left of the dot plot in Figure 4(F1)) compared with that found without the treatment. A comparison of the conditions before and after the hydrogen peroxide treatment indicated that the shift in the Q4/Q1 proportions increased from approximately 10.1 to approximately 31.4 (Figure 4(C1,F1)).

**Figure 5:**
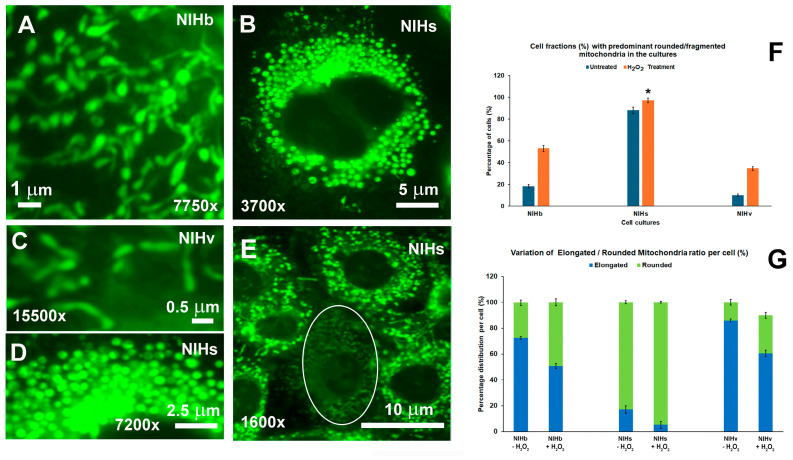
*Details and quantitative aspects of the mitochondrial morphology in NIHb, NIHs, and NIHv*. The high magnification of the areas of the cytoplasm after the DiOC6 fluorochromization allows the morphology of the mitochondria to be observed in detail. Before the hydrogen peroxide treatment, the predominant morphology of elongated mitochondria can be observed in NIHb (**A**) and NIHv (**C**) cells. In the NIHs culture, in which the rounded morphology of the organelles prevails (**B**,**D**,**E**), a cell with a lower fluorescence (within the ellipse) than the other surrounding cells can be observed (**E**). Scale bars and image magnification: in (**A**), 1 μm (7750×); in (**B**), 5 μm (3700×); in (**C**), 0.5 μm (15,500×); in (**D**), 2.5 μm (7200×); and in (**E**): 10 μm (1600×). The graphs in (**F**,**G**) show the quantitative data, obtained after the analysis of the mitochondrial fluorescence images. In (**F**), the values represent the fraction of cells, in the total population examined, in which rounded/fragmented mitochondria prevailed. In (**G**), the data from the evaluation on single cells are represented. In this last case, the values express the mean percentage ratio between the two main types of the mitochondrial morphology, elongated or rounded, present in the cytoplasm. The numerical values represent the means ± SE of triplicate determinations (*n* = 3). In (**F**), in the comparison between untreated cultures, NIHs vs. NIHb cells and NIHv vs. NIHb cells, as well as before and after the treatment of the same culture type (e.g., +H_2_O_2_ NIHb vs. −H_2_O_2_ NIHb), the means were significantly different (*p* < 0.05 after Student’s *t*-test) except for the comparison of +H_2_O_2_ NIHs cells vs. −H_2_O_2_ NIHs cells (asterisk). In (**G**), all the means were significantly different (*p* < 0.05 after Student’s *t*-test).

Fluorescence microscopy images after the fluorochromization of DiOC6 and PI are inserted in the graphs in Figure 4 and allow flow cytometric measurements to be correlated with the distribution and morphology of the mitochondria in cells from different cultures. The magnifications of the cytoplasmic areas shown in Figure 5 allowed us to observe in more detail the prevalent morphology of the mitochondria, which were predominantly elongated in the NIHb (Figure 5A) and NIHv cells (Figure 5C) and rounded in the NIHs cells (Figure 5B,D,E), as detected before the hydrogen peroxide incubation. The quantitative data referring to the counts of the main mitochondrial morphologies, carried out on the fluorescence images (see Section 2 for details), are reported in Figure 5F,G. In the absence of the treatment, in the NIHb culture (inset in Figure 4A and Figure 5A), the fraction of cells in which rounded/fragmented mitochondria prevailed was approximately 18% of the total population examined (Figure 5F). The evaluation, restricted to the cytoplasm of single cells, indicated that mitochondria with a rounded morphology were, on average, approximately 27% of the total chondriome. The remainder showed an elongated morphology (Figure 5G).

The untreated NIHs cultures showed a predominantly rounded mitochondrial morphology (inset in Figure 4B and Figure 5B,D,E) in most of the cells (approximately 88%) (Figure 5F). The evaluation of the single cells indicated that the rounded/fragmented mitochondrial morphology was, on average, approximately 83% of the total mitochondrial content per cell (Figure 5G).

Furthermore, in some areas of the NIHs culture, less fluorescent cells can be observed, indicating some heterogeneity of the DiOC6 labeling (Figure 5E). In NIHv cells, the mitochondria appeared even more elongated than those in the original NIHb culture, especially in the more adherent and enlarged cells, which are typical features of the senescent phenotype (inset in Figure 4C and Figure 5C). In the untreated NIHv culture, the cellular fraction in which rounded/fragmented mitochondria prevailed was only approximately 10% (Figure 5F). The evaluation of the single cell revealed that the rounded morphology was, on average, approximately 14% of the total mitochondria content per cell (Figure 5G).

The hydrogen peroxide treatment induced a general decrease in mitochondrial fluorescence (Figure 4). This effect, together with the increase in the cell thickness, negatively affected the possibility to appreciate the details of organelle features (insets in Figure 4D–F). However, the quantitative analysis of fluorescence images allows us to detect a tendency toward an increase in the number of mitochondria with a rounded/fragmented morphology in all cultures. This appears especially evident in treated NIHb and NIHv cells (insets in Figure 4D,F). In these cultures, the cell fractions in which rounded/fragmented mitochondria prevailed increased, reaching values of approximately 53% and 35%, respectively (Figure 5F). In treated NIHb and NIHv cultures, the single-cell assessment indicated that mitochondria with a rounded morphology were, on average, approximately 49% and 30%, respectively, of the total cytoplasmic content of these organelles (Figure 5G).

Regarding the treated NIHs culture, the cellular fraction in which rounded/fragmented mitochondria prevailed, already with a high incidence in the basal condition, was further increased after the treatment, reaching the value of approximately 97% (Figure 5F). The single-cell evaluation indicated that the elongated morphology was present, on average, for only approximately 5% of the total cellular content of mitochondria (Figure 5G). Furthermore, the fluorescence analysis allowed us to detect that in some microscopic fields, in NIHs and NIHv cells, the lower green mitochondrial fluorescence of DiOC6 correlated with the nuclear red fluorescence due to the propidium iodide uptake (insets in Figure 4E,F).

### 3.5. Detection of Superoxide Anion in Mitochondria Before and After Treatment with Hydrogen Peroxide

**Figure 6:**
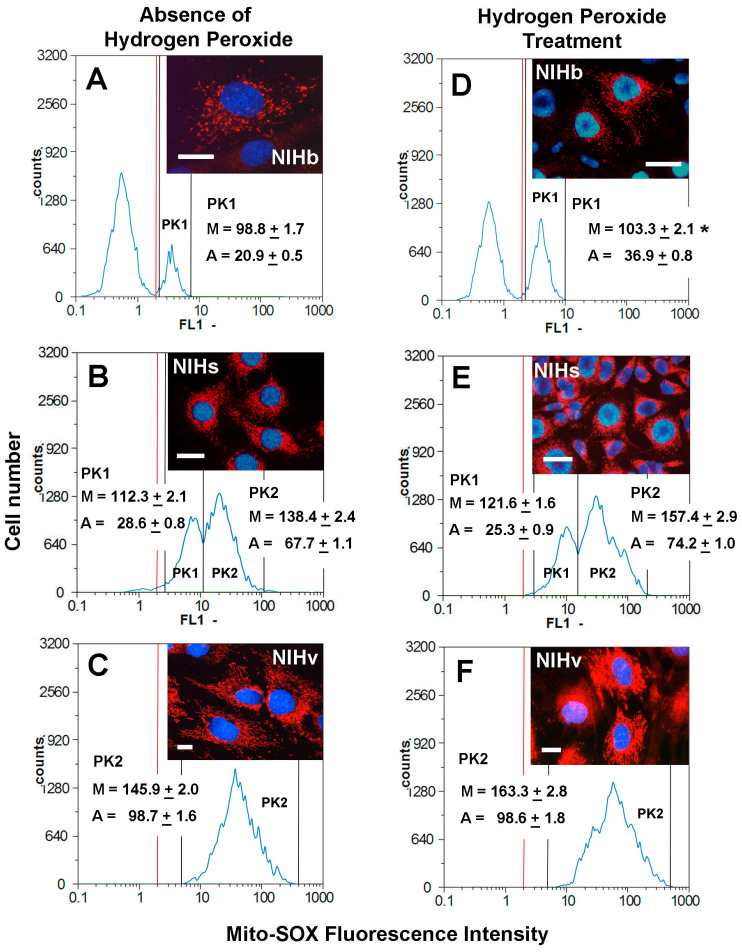
*The microfluorometry and morphology of the superoxide anion labeling before and after the hydrogen peroxide treatment.* Measurements were performed after labeling with MitoSOX Red. The fluorescence intensity values are represented as curves of their frequency distributions. In (**A**–**C**) the results of the measurements before the hydrogen peroxide treatment of NIHb, NIHs, and NIHv cells, respectively, are shown. The curves in (**D**–**F**) refer to the changes induced by hydrogen peroxide in NIHb, NIHs, and NIHv cells, respectively. In the graphs, the threshold (red line) below which the fluorescence intensity values fall between those detected in the background and in the negative labeling cells is shown. M-values indicate the mean MitoSOX Red fluorescence intensity referring to unimodal and/or multimodal distributions. Multimodal patterns express cell fractions with different fluorescence characteristics. A-values indicate the percentage of cells with specific fluorescence characteristics and are an expression of peak areas, compared to the whole area subtended by the curves. The numerical values were calculated on three independent determinations (*n* = 3) and represent the mean (±SE). With the exception of the M-value marked with an asterisk (*), the other values reported were statistically significant (*p* < 0.05 after Student’s *t*-test), both when comparing untreated cultures [NIHs (**B**) and NIHv (**C**) vs. NIHb (**A**)] and, within the same culture, between treated and untreated cells (NIHb: (**D**) vs. (**A**); NIHs: (**E**) vs. (**B**); and NIHv: (**F**) vs. (**C**)). In the insets, the red fluorescence of MitoSOX allows us to appreciate the distribution of the superoxide anion in the cytoplasm of the cells. In (**A**–**C**), micrographs before the hydrogen peroxide treatment of NIHb, NIHs, and NIHv cells, respectively, are shown. In (**D**–**F**) the situation after the incubation with hydrogen peroxide in NIHb, NIHs, and NIHv cells, respectively, is shown. Staining the nuclei with Hoechst 33342 (blue fluorescence) helps to identify the location of cells with a reduced or absent labeling of MitoSOX Red (**A**,**D**). Scale bars in (**A**,**C**,**D**,**F**): 5 μm; in (**B**,**E**): 10 μm.

The detection and measurement of ROS formation in intracellular compartments can be an important analytical contribution to characterize the cultures used here under different experimental conditions. In general, mitochondria have been identified as a major source of superoxide anion production at the cellular level. To determine whether the variation in the levels of this reactive species could be linked to changes in the metabolism and morphology of the mitochondria, we attempted to quantify, by means of microfluorometry, the formation of the superoxide anion. From a methodological point of view, several techniques for detecting the superoxide anion at the mitochondrial level have been developed for over ten years. Among these, we chose an assay based on the use of the fluorogenic compound MitoSOX Red. To detect relative differences in the formation of the mitochondrial superoxide anion, the fluorescence emitted after the labeling with MitoSOX Red was analyzed by both fluorescence microscopy and microfluorimetry (for details see Section 2). Both morphological and quantitative results indicated different situations emerging after labeling the cultures under different experimental conditions (Figure 6).

In the untreated NIHb culture, the cells showed a mainly weak positivity to labeling. Only in a cell fraction, on average 20.9%, was it possible to measure significantly more pronounced fluorescence signals (inset in Figure 6A), with mean intensity values of 98.8 (Figure 6A). After the treatment with hydrogen peroxide, no statistically significant change in the mean fluorescence intensity was detected in NIHb cells compared to the untreated condition. However, the percentage of cells with a higher labeling level increased to a mean value of 36.9% (Figure 6D).

In the culture of NIHs, in the absence of the treatment, all cells showed clear labeling (inset in Figure 6B). After the measurements, the fluorescence intensity values showed a bimodal frequency distribution pattern (Figure 6B). The microfluorometric data thus made it possible to identify two cell fractions with mean values of 112.3 (28.6% of cells on average) and 138.4 (67.7% of cells on average). After the incubation of the NIHs culture with hydrogen peroxide, the microfluorometric data confirmed the persistence of some labeling heterogeneity (Figure 6E). However, the two cell fractions, with lower and higher fluorescence, showed an increase in mean intensity values, which were 121.6 (25.3% of cells on average) and 157.4 (74.2% of cells on average), respectively (Figure 6E).

In the untreated NIHv culture, almost all cells (98.8% of cells on average) were positive for labeling (inset in Figure 6C), with the highest mean fluorescence intensity value (145.9), which was higher than that measured in all fractions of the other cultures (Figure 6A,B). The high basal level of the superoxide anion production in NIHv cells further increased after the incubation with hydrogen peroxide. Almost all cells (98.6% of cells on average) showed a mean fluorescence intensity value of 163.3 (Figure 6F). In NIHv cells, both before and after the treatment with hydrogen peroxide, the dispersion of fluorescence signals along the abscissa is indicative of a significant heterogeneity in the MitoSOX Red labeling.

### 3.6. Lysosome Content/Activity, Lipofuscin Accumulation, and Activity of SA-β-Galactosidase Before and After Hydrogen Peroxide Treatment

#### 3.6.1. Lysosomal Compartment and Lipofuscin Accumulation

The fluorescent probe Lysotracker Red (LTR) and the metachromatic fluorochrome Acridine Orange (AO) were used to analyze the lysosomal compartment under cell viability conditions. The chemical–physical properties of these fluorochromes allow them to accumulate in lysosomes and acid vesicles, and the fluorescence emission depends on the internal pH that correlates with their functional activity.

Acridine Orange, used according to appropriate protocols (see Section 2 for details), can also provide more information on the involvement of lysosomes and acidic vesicles in relation to autophagic events. The labeling with Lysotracker Red was analyzed by microfluorimetry and fluorescence microscopy, which made it possible to obtain information on the variation in the lysosome content/activity and its distribution in the cells of the cultures under different experimental conditions.

**Figure 7:**
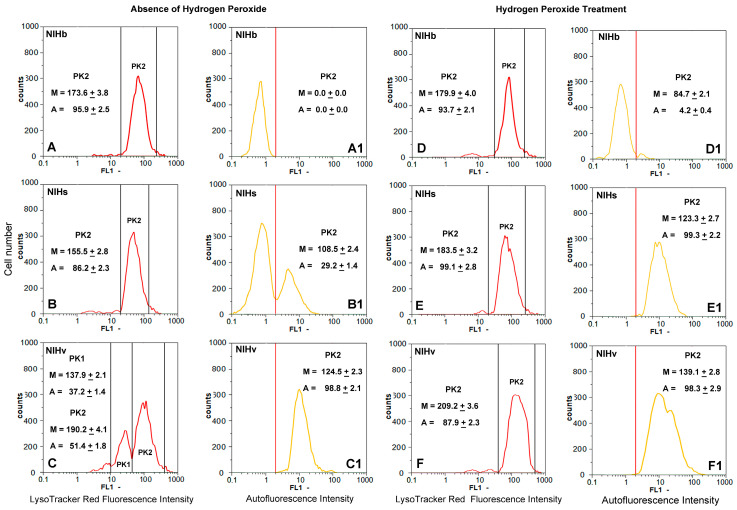
*The microfluorometry of the lysosome labeling and the autofluorescence of the lipofuscin before and after the hydrogen peroxide treatment*. The cells were fluorochromized with Lysotracker Red for labeling the lysosomes (**A**–**F**). After measuring the red fluorescence, the yellow green autofluorescence of the lipofuscin (**A1**–**F1**) was detected in the same cells. The two different light emissions were obtained using different excitation wavelengths (see Section 2 for details). The fluorescence intensity values were represented as curves of their frequency distribution. In (**A**,**A1**,**B**,**B1**,**C**,**C1**) the results of measurements before the hydrogen peroxide treatment of NIHb, NIHs, and NIHv cells, respectively, are shown. The curves in (**D**,**D1**,**E**,**E1**,**F**,**F1**) refer to the changes induced by the incubation with hydrogen peroxide in NIHb, NIHs, and NIHv cells, respectively. M-values indicate the mean intensity of the lysosomal fluorescence or lipofuscin autofluorescence referring to unimodal and/or multimodal distributions. Multimodal trends express cell fractions with different characteristics. A-values indicate the percentage of cells with specific fluorescence characteristics and are an expression of peak areas, compared to the whole area subtended by the curves. The numerical values were calculated on the basis of three independent determinations (*n* = 3) and represent the mean (±SE). All reported mean values were statistically significant (*p* < 0.05 after Student’s *t*-test), both when comparing untreated cultures [NIHs (**B**) and NIHv (**C**) vs. NIHb (**A**)] and, within the same culture, between treated and untreated cells (NIHb: (**D**) vs. (**A**); NIHs: (**E**) vs. (**B**); and NIHv: (**F**) vs. (**C**)).

In the absence of the hydrogen peroxide treatment, the examination of the microfluorometric frequency curves of the original NIHb culture showed, in 96.9% of the cells on average, a homogeneous fluorescence distribution with a mean value of 173.6 (Figure 7A). After the incubation with hydrogen peroxide, a very limited increase in the NIHb culture was measured: 93.7% of the cells on average showed a mean fluorescence intensity value of 179.9 (Figure 7D). In the absence of the treatment, NIHs cells showed a significantly lower lysosomal content/activity than NIHb cells under the same conditions. The analysis of the curve of the frequency distribution of the NIHs cells indicated a mean value of the lysosome fluorescence intensity of 155.5, in 86.2% of cells on average (Figure 7B). In the NIHs culture, after the treatment with hydrogen peroxide, a significant increase in the lysosomal content/activity was detected: in almost all cells (99.1% of cells on average), a mean fluorescence intensity value of 183.5 was measured (Figure 7E). In the NIHv culture, in the absence of the hydrogen peroxide treatment, the examination of the frequency distribution curves (Figure 7C) made it possible to identify at least two distinct cell fractions, with a different percentage incidence and a different lysosomal content/activity. The mean fluorescence intensity value for the fraction of cells with the lowest percentage incidence (37.2% on average) was 137.9. The predominant fraction (51.4% on average) was represented by NIHv cells with the highest lysosomal content/activity, with a mean fluorescence intensity value of 190.2 (Figure 7C), which was higher than that found in the other untreated cultures (Figure 7A,B). After the treatment with hydrogen peroxide, the two NIHv cell fractions displayed in the untreated culture (Figure 7C) were no longer identifiable (Figure 7F). Only one prevailing fraction (87.9% on average) was detected, in which the highest lysosomal content/activity was expressed by a mean fluorescence intensity value of 209.2 (Figure 7F).

**Figure 8:**
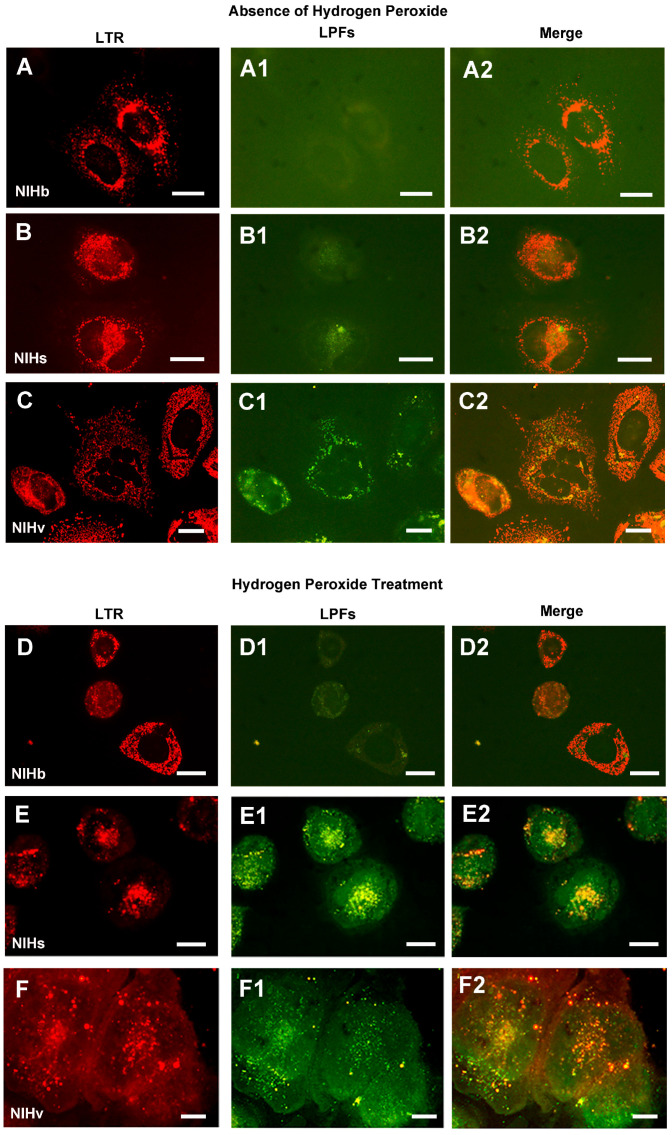
*The morphology of lysosomes and the autofluorescent inclusion labeling and their co-localization in the cytoplasm.* The fluorescence microscopy of the lysosome labeling with Lysotracker Red (LTR) before (**A**–**C**) and after the hydrogen peroxide treatment (**D**–**F**). In the same cells, under adequate conditions of excitation (see Section 2 for details), it is possible to obtain the cytoplasmic autofluorescence of lipofuscin (LPF) inclusions, before (**A1**–**C1**) and after the hydrogen peroxide treatment (**D1**–**F1**). In order to evaluate the co-localization of the lipofuscin accumulation granules (green fluorescence) with the lysosomes (red fluorescence), in (**A2**–**F2**), the merged images of the two fluorescence signals in the respective experimental conditions are shown. The overlap of the two fluorescence signals results in the yellow color which appears in the form of dots or aggregates. Scale bars: 5 μm.

In the absence of the treatment, in NIHb and NIHs cells, the lysosomal fluorescence appeared to be predominantly located in perinuclear areas (Figure 8A,B), contrary to what was observed in NIHv cells (Figure 8C).

The correlation between the labeling of lysosomes with Lysotracker Red and the emission of autofluorescence due to the accumulation of lipofuscin inclusions revealed the following. In the untreated NIHb reference culture, the morphological analysis revealed no substantial autofluorescence emissions of lipofuscin (Figure 8(A1)). From a microfluorometric point of view, signals with values close to those of the background brightness were detected (Figure 7(A1)). Following the treatment with hydrogen peroxide, very limited autofluorescent spots were detected in only a few NIHb cells (Figure 8(D1)). This microscopic observation was confirmed by the microfluorometric analysis, which resulted in detecting a mean autofluorescence intensity value of 84.7 in just over 4.2% of the cells on average (Figure 7(D1)).

In the untreated NIHs culture, the morphological analysis revealed limited autofluorescence in part of the cells (Figure 8(B1)). The quantitative analysis identified a cell fraction of 29.2% on average, in which a mean autofluorescence intensity value of 108.5 was measured (Figure 7(B1)). After the hydrogen peroxide treatment of the NIHs culture, the morphological analysis revealed that lipofuscin autofluorescence was present in almost all cells and appeared, with a certain frequency, as aggregates (Figure 8(E1)). The quantification of these aggregates is presented in Table 1 as the LPAI (Lipofuscin Aggregates Index) obtained from the comparison between the cytoplasmic area occupied by the lipofuscin aggregates and the total cell area. In treated NIHs cells, microfluorometric measurements indicated an increase in the autofluorescence intensity, expressed by a mean value of 123.3 (Figure 7(E1)).

The cells of the untreated NIHv culture were generally all autofluorescent; microfluorometric measurements revealed a mean intensity value of 124.5 (Figure 7(C1)). The morphological examination showed, in some microscopic fields, heterogeneity in the distribution and size of the lipofuscin accumulation granules and their aggregates (Figure 8(C1); Table 1).

After the incubation of NIHv cells with hydrogen peroxide, microfluorometric measurements indicated an increase in autofluorescence with a mean intensity value of 139.1 in almost all cells (Figure 7(F1); for the morphology see also Figure 8(F1). The quantitative data from treated NIHv cells indicate an increase in the cytoplasmic area occupied by lipofuscin aggregates (Table 1). This table also shows the cytoplasmic co-localization index between the LTR fluorescence and LPFs autofluorescence.

The values of the lysosomal red fluorescence aggregation index (LYAI), the lipofuscin green autofluorescence aggregation index (LPAI), and the co-localization index of the two fluorescence signals (COLI) are reported. The LYAI and LPAI percentage values were normalized by comparing them to the total area of the individual cells measured. The COLI values, obtained by the measurements of the red and green fluorescent areas (yellow spots), were normalized considering the total cell area and were expressed as a percentage of the red fluorescent lysosomal compartment.

The values were calculated based on three independent determinations (n = 3) and represent the mean (±SE). For each measured index, all the mean values were significantly different (*p* < 0.05 after Student’s *t*-test), when comparing untreated NIHs and NHIv vs. untreated NIHb cultures, as well as before and after the treatment of the same culture type (e.g., +H_2_O_2_ NIHb vs. −H_2_O_2_ NIHb).

The level of the overlap of the two light emissions, resulting in a yellow color in the merged fluorescence images (Figure 8(A2–F2)), was determined after comparing the size of the yellow cytoplasmic areas and the red LTR fluorescence of the entire lysosomal compartment. Therefore, the numerical values, reported in the fourth column of Table 1, express the percentage of their overlap. The yellow spots are morphologically identifiable as single or aggregated spots of a heterogeneous size (Figure 8(A2–F2)).

In the absence of the treatment, the lack of lipofuscin inclusions in NIHb cells (Figure 8(A1)) does not allow us to detect any co-localization (Figure 8(A2)). The incubation of this culture with hydrogen peroxide determines the appearance of rare green autofluorescence spots with limited co-localization values (Figure 8(D2); Table 1).

In particular, in NIHs and NIHv cells, and under specific experimental conditions, the fluorescence microscopy examination allowed us to establish that the accumulation sites of lipofuscin inclusions partially co-localize with lysosomal labeling (Figure 8(B2,C2,E2,F2)). In the NIHs culture in basal conditions, the limited presence of cytoplasmic autofluorescence (Figure 8(B1)) resulted in a low level of co-localization compared with the total red fluorescence of lysosomes (Figure 8(B2); Table 1). Conversely to treated NIHb cells, the significant presence of lipofuscin granules induced in NIHs cells by hydrogen peroxide (Figure 8(E1)) allows the detection of a co-localization level of more than 22% on average (Figure 8(E2); Table 1).

In untreated NIHv cells, a mean co-localization level of approximately 26% was recorded (Figure 8(C2); Table 1), which was the highest compared with that in other cultures. After the incubation with hydrogen peroxide, NIHv cells showed a significant increase in co-localization with values exceeding 31% on average (Figure 8(F2); Table 1). Especially after treatment, almost all the larger red fluorescence spots (Figure 8F) tended to co-localize with the emission of green autofluorescence (Figure 8(F1)), thus producing a more intense yellow color (Figure 8(F2)).

#### 3.6.2. Detection of SA-β-Galactosidase Activity Before and After Hydrogen Peroxide Treatment

**Figure 9:**
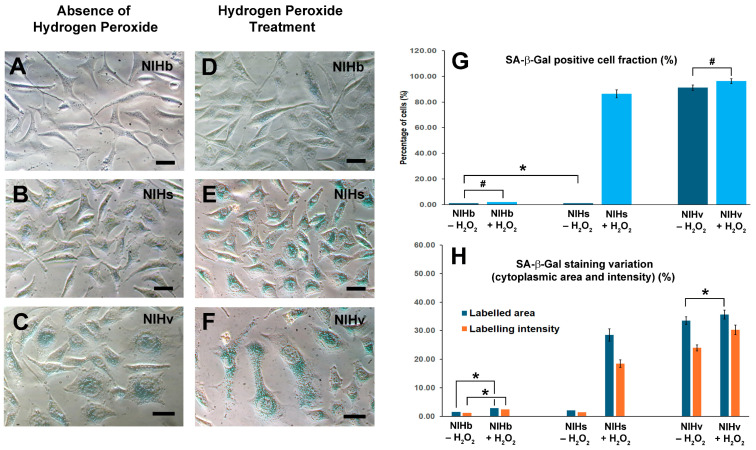
*Morphological and quantitative aspects of SA-β-Gal staining in NIHb, NIHs, and NIHv cells, before and after the treatment with hydrogen peroxide*. Bluish-green cytoplasmic staining indicates positivity for the chromogenic reaction, revealing senescence-associated-β-galactosidase (SA-β-Gal) activity. In (**A**–**C**), NIHb, NIHs and NIHv cells before hydrogen peroxide treatment are shown; in (**D**–**F**), NIHb, NIHs and NIHv cells after hydrogen peroxide treatment are shown, respectively. Scale bars: 10 μm. In (**G**,**H**), the graphs show quantitative data of chromogenic reaction. In (**G**), the plotted values refer to the percentage fractions made up of cells with a bluish-green color, in comparison with the totality of the cell population examined. In (**H**), the variation in cytoplasmic staining is presented both in terms of labeled area and staining intensity; the values detected (stained areas and intensity) in the individual cells were normalized taking into account the respective total cell areas (See Section 2 for details). In (**G**,**H**), the numerical values, expressed as percentages, represent the mean ± SE of triplicate determinations (*n* = 3). In (**G**), in the comparison between untreated NIHs vs. NIHb cells, the means are not significantly different (* asterisk), whereas the value of NIHv vs. that of NIHb cells is significantly different (*p* < 0.05 after Student’s *t*-test). Within the same culture, before and after treatment, the means were significantly different only for NIHs (+H_2_O_2_) vs. NIHs (−H_2_O_2_). The means were not significant different (# hashtag) for NIHb (+H_2_O_2_) vs. NIHb (−H_2_ O_2_) and NIHv (+H_2_O_2_) vs. NIHv (−H_2_O_2_). (*p* > 0.05 after Student’s *t*-test). In (**H**), the comparison between the means referring to both the extension of the stained areas (blue columns) and the labeling intensity (orange columns), indicated, within the same culture, non-significant values (* asterisk) between the conditions after (+H_2_O_2_) and before (−H_2_O_2_) treatment, for NIHb cells (both labeled area and staining intensity). For NIHv cells, only the comparison of the labeled areas was not significant different (*p* > 0.05 after Student’s *t*-test).

The detection of senescence-associated-β-galactosidase (SA-β-Gal) activity at pH 6.0 is a commonly used biomarker method for the identification and characterization of senescent cells. The evaluation of this enzyme activity applied to our experimental model may offer complementary information to interpret the cellular dynamics that emerge when comparing cultures analyzed both under basal conditions and after the hydrogen peroxide treatment. From a methodological point of view, the enzymatic activity of SA-β-Gal is assessed by means of a chromogenic reaction. Before the treatment with hydrogen peroxide, occasional weak staining was detected in rare NIHb cells (Figure 9A). In the NIHs culture, the cells were found to be negative (Figure 9B). The labeling of SA-β-Gal was clearly evident only in the NIHv culture, in which all the cells were positive (Figure 9C).

**Figure 10:**
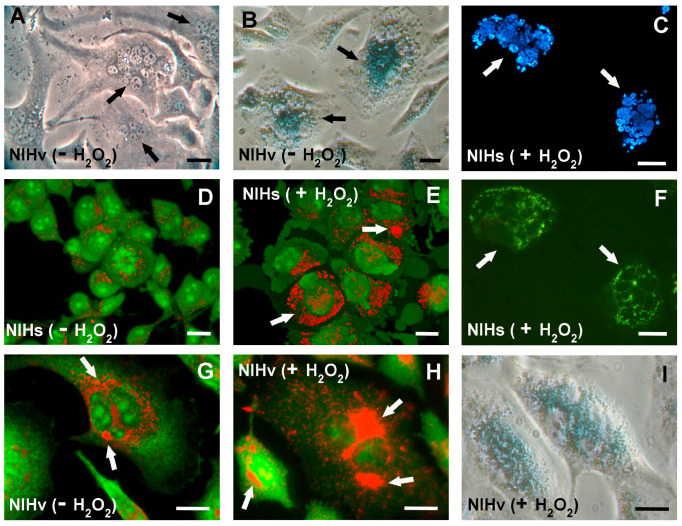
*Nuclear abnormalities and cytoplasmic lysosomal/autophagic activity of untreated and treated NIHv and NIHs cells.* The micrographs in (**A**,**B**,**G**–**I**) refer to NIHv cells, while the micrographs in (**C**–**F**) refer to NIHs cells. In untreated NIHv cells (**A**,**B**), conditions of multinuclearity/mitotic catastrophes can be observed (arrows); in the micrograph in (**B**), the intense bluish-green marking for SA-β-Gal tends to mask the presence of nuclei/micronuclei in NIHv cells (arrows). In (**C**), similar nuclear situations can be detected in NIHs cells treated with hydrogen peroxide after DNA staining with Hoechst 33342 (arrows). In (**F**), the autofluorescence of lipofuscins can be observed in the same NIHs cells whose nuclear situation is visible in (**C**) (arrows). After AO staining, the red florescence in (**D**,**E**) indicates the acidic compartment in the NIHs cells before (**D**) and after (**E**) the hydrogen peroxide treatment. In the micrograph in (**E**), the staining reveals the intense labeling (red fluorescence) of lysosomes and autophagosomes (arrows). The red fluorescence in (**G**,**H**) indicates the acidic compartment in the NIHv cells before (**G**) and after (**H**) the hydrogen peroxide treatment; the increased red fluorescence of lysosomes and autophagosomes, around the nuclei (**H**), is observed after the treatment (arrows). Under this experimental condition, the intense bluish-green labeling for SA-β-Gal can be detected near and above the nuclear area of NIHv cells (**I**). Scale bars in (**A**,**C**,**D**,**F**–**I**): 10 μm; in (**B**,**E**): 5 μm.

The chromogenic reaction was particularly intense in cells with extensive cytoplasm and was markedly adherent to the substrate. The details shown in Figure 10B allow us to highlight how widespread labeling in these NIHv cells often correlates with multinuclear conditions and mitotic catastrophe events.

After the treatment with hydrogen peroxide, a very weak positivity, slightly higher than that of the untreated condition, was detected in only a few cells of the NIHb reference culture (Figure 9D,G,H). In contrast, the NIHs culture showed a good positivity of the chromogenic reaction in approximately 90% of the cells (Figure 9E,G,H). In the senescent NIHv culture, the incubation with the oxidizing agent did not produce any significant quantitative differences (Figure 9F–H) compared with the situation described in the untreated condition (Figure 9C,G,H). However, after the treatment with hydrogen peroxide, the comparison between NIHs and NIHv cells allowed us to detect, in the latter, a greater cytoplasmic labeling, both in terms of the area involved and the bluish-green intensity (Figure 9F–H). This was particularly evident around the nuclei and micronuclei of the largest and most adherent NIHv cells (Figure 10I).

#### 3.6.3. Detection of Autophagic Events Before and After Hydrogen Peroxide Treatment

As reported in Section 2, the Acridine Orange probe can be used as a marker of autophagy in vital stains. In this way, it is possible not only to visualize acidic intracellular compartments but also to quantify their size, which can increase in relation to events, such as autophagy. Cytoplasmic areas involved in autodegradative processes, which emit a wide and intense red fluorescence, were measured with dedicated software applied to microscopy images. In this way, it was possible to obtain a quantitative estimate of autophagic events. The overall extent of the red fluorescence was correlated with the cell size, which was estimated by measuring the surface area occupied on the growth substrate, expressed by the weak cytoplasmic green fluorescence. In this way, an indirect parameter of autophagic activity called the MAI (Mean Autophagic Index) was obtained. The results were expressed as the percentage of MAI-positive cells. A positivity threshold of 2.30 was established by considering the MAI value measured most frequently in untreated NIHb cells. Thus, all cells in the different experimental conditions, in which an MAI value greater than 2.30 was detected, were considered autophagic. The values of the percentage incidence of positive cells and the MAI measured before and after the hydrogen peroxide treatment are reported in Table 2.

In the untreated NIHb culture, the percentage of autophagy-positive cells with more prominent cytoplasmic red fluorescent aggregates was 4.91% on average. An MAI of 2.70 was found in these cells (Table 2). The incubation of the NIHb culture with hydrogen peroxide resulted in an increase in the number of autophagy-positive cells, which were 7.76% on average, and the measured MAI was 3.31 (Table 2).

In the absence of the treatment, the comparison between the NIHb culture and the NIHs culture showed, for the latter, a higher autophagic activity, detected in 6.05% of cells on average. In these untreated NIHs cells, the MAI reached a value of 3.29 (Table 2), which was correlated with a slight increase in the size of the red fluorescent aggregates in the cytoplasm (Figure 10D). The incubation of the NIHs culture with hydrogen peroxide resulted in an increase in larger red fluorescent areas in the cytoplasm (Figure 10E). The occurrence of autophagic processes was detected in 15.49% of the cells on average (Table 2). The MAI value was 7.01 (Table 2). In the untreated NIHv culture, the MAI of 8.26, which was higher than that in the other cultures, was measured in 23.21% of the cells on average (Table 2). Morphologically, some labeling heterogeneity was observed between cells. In several cells, the most extensive cytoplasm showed a diffuse granularity accompanied by areas where the red fluorescence was concentrated (Figure 10G). The incubation of the NIHv culture with the oxidizing agent induced an increase in the MAI, which reached a value of 18.52 (Table 2). In this culture, an incidence of 37.27% of cells involved in autophagic events was measured (Table 2). An increase in fluorescent red granulations that tended to aggregate in the cytoplasm was also observed (Figure 10H).

To obtain an overall view of the cellular aspects compared between the different cultures examined, before and after the treatment with hydrogen peroxide, the main data are summarized in Table 3 below.

The main cellular parameters examined in NIHb, NIHs, and NIHv cultures, before and after the hydrogen peroxide treatment, are reported. The values are extrapolated from the graphs and tables above. In the case of the cyto-/microfluorimetric measurements, the values shown refer to the highest fluorescence intensities (PK2 peak).

## 4. Discussion

### 4.1. Cell Cycle Progression Before and After Hydrogen Peroxide Treatment

As the knowledge of cellular senescence has progressed, the acquisition of information on the programs of its expression has revealed complex situations, partly linked to the heterogeneity of the phenotypes involved. Paradoxically, this has made it more difficult to assess certain cellular aspects related to the manifestation of the phenomenon in different study models. In our experimental model, the phenotypic characteristics of NIHb, NIHs, and NIHv cells, associated, respectively, with a state of normality, transformation, or already acquired senescence, seemed to determine a different sensitivity to the stress conditions induced by the incubation with the same concentration of hydrogen peroxide. In the absence of the treatment, compared with the NIHb culture, the NIHs culture showed greater proliferative activity, with a higher percentage of cells in the S phase of the cell cycle. If we accept that the NIHs cells reached a condition close to the transformed state, the acquired proliferative characteristics could be related to the metabolic changes resulting from the altered redox state influenced by the increase in intracellular ROS. The use of the fluorogenic indicator MitoSOX substantially confirmed this hypothesis, allowing the detection of, morphologically and quantitatively, an increase, at the mitochondrial level, in the production of the superoxide anion, compared to the NIHb reference culture. As already reported in previous works on transformed fibroblasts, significant amounts of ROS were correlated with increased proliferative activity [24]. On the other hand, the incubation of the NIHs culture with hydrogen peroxide resulted in a greater slowdown of the G1-S transition, with a marked decrease in S-phase cells and an accumulation in the G1 phase of the cell cycle. The increased production of the superoxide anion detected after the treatment could be representative of additional oxidative stress conditions that influence the regulation of the cycling activity of NIHs cells, with an anti-proliferative effect. Previous studies have shown that, depending on the phenotypic characteristics of the cells, hydrogen peroxide, like other oxidizing agents, can induce variable effects. As a consequence of changes in the redox state, some tumor cells may be hindered in proliferation and start to die, while others may be stimulated in growth, with the activation of transcription factors or the inhibition of tumor suppressor genes [13].

In models consisting of immortalized keratinocytes, in which oxidative stress was induced by hydrogen peroxide, or UVB-irradiation, genes encoding peptides with antioxidant activity with effects on different cellular functions have been identified [25].

In our experimental model, in the original NIHb culture, the more limited effects induced by hydrogen peroxide on proliferative activity were related to the more moderate increase in the superoxide anion production. Thus, the specific antiproliferative effect revealed by the comparison of NIHb and NIHs cells could be attributable to the different magnitude of the treatment-induced change in the redox state compared with basal conditions. In rapidly proliferating transformed cells, the basal concentration of hydrogen peroxide is usually higher than that in normal cells [13].

However, in both NIHb and NIHs cultures, the changed redox state could influence the G1 restriction point of the cell cycle through the regulation of the activity of CDKs and their inhibitors [26]. The greater number of NIHs cells in the S phase, detected under basal conditions, would have made them more sensitive to hydrogen peroxide-induced oxidative stress. Intracellular hydrogen peroxide levels and their modulation may represent a possible target of antiproliferative chemotherapy activity. Recent studies reported, for example, that bioactive cisplatin can increase the level of endogenous hydrogen peroxide. The latter represents a substrate which, through the catalysis of biochemical reactions, can be transformed into highly reactive and toxic hydroxyl radicals with anti-tumor activity [27].

The different responses to the cytostatic action, which emerged when NIHb and NIHs cells were compared, were also related to the different expression dynamics of the SA-β-galactosidase enzyme activity. Under basal conditions, both cultures were substantially negative for the chromogenic reaction. After the incubation with hydrogen peroxide, the labeling appeared absent or very weak in the NIHb culture, whereas it was evident in the NIHs culture. It has already been reported that hydrogen peroxide is also able to induce senescence and SA-β-galactosidase positivity in tumor cell lines [28,29]. In the already senescent NIHv culture, the labeling of SA-β-Gal, which is very evident under basal conditions, does not appear substantially changed after the incubation with hydrogen peroxide. However, even in NIHv cultures, the effects induced by the oxidizing agent on the cell cycle may depend in part on the cytokinetic characteristics and the specific cellular redox state detected under basal conditions. Compared with the reference NIHb culture, the flow cytometric analysis of untreated NIHv cells revealed an imbalance between cell cycle phases. In this culture, in addition to the low percentage incidence of cells in the S phase, an accumulation of cells in the G2/M phase of the cell cycle was detected. Thus, in the untreated NIHv cultures, the predominant part of the cells showed little or no proliferative capacity. A limited fraction of cells in the NIHv culture, less than 10% of the total, were still able to enter the S phase of the cell cycle. The NIHv cells that still had some proliferative activity were characterized by their small size, while the larger cells, with extended and flattened cytoplasm, appeared to have little or no involvement. In these larger cells, the higher adhesive properties were often correlated with multinuclearity. The occurrence of such nuclear conditions, which were detected in the NIHv culture even in the absence of the treatment, could be the consequence of difficulties in the division processes, as also demonstrated by mitotic catastrophe events and the presence of micronuclei. In cells with these cytoplasmic and nuclear features, positivity for the SA-β-Gal chromogenic reaction was particularly widespread and more intense than that in the other cells in the culture. This cytochemical characteristic of NIHv cells is consistent with previous reports in which the induction of nuclear abnormalities and mitotic catastrophe have been associated with cellular senescence and SA-β-Gal positivity [30]. The greater expression of this enzyme activity in NIHv cells appeared to be correlated with a state of greater oxidative stress, also indicated by the higher production of the superoxide anion detected already under basal conditions. This cellular state could influence the events involved in cell cycle regulation, inducing its slowing down and the evident decrease in S-phase cells in most untreated senescent NIHv cells. However, given that a large percentage of cells accumulate in the G2/M phase of the cell cycle, more than one mechanism could be involved in the decline in the proliferative activity. At least part of the cells blocked in the G2/M phase would derive from those still able to pass the S phase of the cell cycle and proceed through mitosis, with possible malfunctions underlying this process. From a cytometric point of view, in the slow-proliferating and untreated NIHv culture, the high percentage of G2/M signals could derive from repeated premitotic and mitotic blockages in those cells that, in successive cell cycles, manage to pass the S phase. This cytokinetic interpretation would also be supported by the frequency with which binucleated cells were detected. On the other hand, the presence of multinucleated cells (even with micronuclei) could be compatible with the fluorescence signals to the right of the G2/M peak.

Therefore, regardless of the hydrogen peroxide treatment, oxidative stress related to the senescence conditions in NIHv cells could play a role in promoting not only an inefficiency of the G1/S transition but also abnormalities and/or the arrest of the mitotic process. Previous work has shown that both exogenous and endogenous ROS can influence normal mitotic spindle formation, resulting in abnormal chromosomes even in tumor cells [31]. Normally, cell division processes are regulated by the intracellular redox environment [31], and ROS production varies during cell cycle progression, reaching its maximum during mitosis. The mitotic blockade can lead to a further increase in ROS [32].

In our cell model, the treatment with hydrogen peroxide induced a decrease in the percentage of NIHv cells in the G2/M phase of the cell cycle. This could be partly a consequence of the reduction in the number of cells crossing the G1-S transition (which further enriches the accumulation of cells in the G1 phase) and partly due to the autophagic cell death events involving larger, often binucleated cells that characterize NIHv cultures. With respect to the comparison of hydrogen peroxide-induced effects on the cell cycle progression of transformed NIHs cells and senescent NIHv cells, the respective changes appeared to be dependent on the proliferative activity expressed under basal conditions by the two cultures. Furthermore, regardless of the difference in the basal expression of the SA-β-Gal activity, which was detected only in the case of the senescent NIHv culture, both NIHs and NIHv cell types showed, after the treatment, an increase of the same order in the level of the superoxide anion production. At the same time, NIHs cells became positive for SA-β-Gal, while NIHv cells showed more limited changes in the chromogenic reaction. These data seem to indicate that, in cells with different biological properties, the dynamics of senescence expression can be regulated through possible alternative pathways. In NIHv cells, the replicative senescence program manifested under basal conditions, as in other models [33], would be slightly altered by the treatment with the oxidizing agent, despite the increase in the superoxide anion production detected. On the other hand, in NIHs cells, a similar increase in the ROS, caused by the treatment, would be able to induce senescence as a possible consequence of a more significant overall change in the redox state, compared with that under basal conditions. However, it should still be emphasized that, in relation to the characteristic of the fast cell proliferation, the endogenous hydrogen peroxide level of transformed cells is higher than that of non-transformed cells [13]. This feature could explain the greater reactivity of NIHs cells to the hydrogen peroxide treatment, which would induce additional oxidative stress due to the intracellular increase in reactive oxygen species. The result would be the establishment of a senescence condition that shows similarities to what may be defined as “premature” [33]. Under this condition, cells usually display biomarkers common to replicative senescent cells. However, it must be underlined that the nature of senescence in tumor biology involves a series of complex and multifaceted aspects. In the case of tumors, the detection of SA-β-Gal activity still plays an important role, although other markers such as p16 can be taken into consideration to better characterize the process [34]. In our experimental model, in addition to the positivity shown by NIHs cells, after the hydrogen peroxide treatment, more than one cellular aspect, discussed below, converges, in our opinion, in supporting the acquisition, in these cells, of a phenotype showing characteristics of senescence.

In our experimental model, hydrogen peroxide may have a different influence on the induction/modulation of the senescence state, depending on the cytokinetic properties and metabolic condition exhibited by the cells in the different cultures.

### 4.2. Content/Activity and Morphology of Mitochondria Before and After Treatment with Hydrogen Peroxide

In the absence of the treatment, the different proliferative activities detected in the NIHb, NIHs, and NIHv cells was associated with a cell morphology indicative of a different abilities to adhere to the growth substrate. Compared with the NIHb reference culture cells, the NIHs and NIHv cells showed lower or higher adhesion properties, respectively. These characteristics could reasonably be reflected in a different organization of the cytoskeleton, which, especially in the microtubular component, could influence the morphology and size of the mitochondria, favoring their fragmentation or fusion. Less adherent NIHs cells showed a subdivision of the chondriome with a predominantly rounded mitochondrial morphology, which is an alternative to the elongated structure found mainly in NIHb and NIHv cells. Works in recent years have highlighted the complexity of the regulatory networks that couple mitochondrial dynamics to the increased rate of cell proliferation [35]. During the cell cycle progression, changes in the metabolic state correlate with both the total mass of mitochondria and their morphology. The latter is regulated by a set of proteins that can lead to the fusion or fission of these organelles [36]. It remains to be fully defined whether these morphological changes can be reflected in significant functional modifications and have a relationship with cytokinetic aspects. According to some works, the transient condition of mitochondria in the accentuated fusion state appears to be crucial for the increase in cyclin E levels required for the entry into the S phase. On the other hand, alterations in the fusion capacity of mitochondria have been shown to result in the activation of G1/S cell cycle checkpoints with an arrest in the G1 phase due to reduced cyclin E levels [36]. However, it should be emphasized that the prolongation of the diffuse mitochondrial fusion condition over time has been shown to cause a delay in the entry into the S phase, as well as blockage of G2/M and aneuploidy, as a consequence of the cyclin E accumulation. Therefore, mitochondrial fragmentation would be necessary for cells to enter mitosis [36]. Based on this evidence, the relationships between the organization and function of the mitochondrial compartment and changes in cell cycle progression may play a crucial role in the onset and regulation of senescence and cellular transformation [37,38]. In our experimental model, the interdependence between the proliferative activity and mitochondrial content/activity could influence the expression dynamics of the different cellular phenotypes and therefore also regulate the heterogeneity of the subpopulations constituting the cultures examined. In this context, the assessment of the accumulation capacity of the vital fluorochrome DiOC6 in the mitochondrial matrix may provide some information. The intensity of the measured fluorescence depends on the transmembrane potential ΔΨm and is thus correlated with both the degree of the functional activity and the mass of the mitochondria. For these reasons, we expressed the fluorescence of DiOC6, assessed cytometrically, as the mitochondrial content/activity of the cells. This parameter showed a certain heterogeneity between the cell populations constituting mainly NIHs and NIHv cultures, where the histograms of the fluorescence intensity expressed a bimodal trend under the different experimental conditions.

In the absence of the hydrogen peroxide treatment, the highest mean value of the fluorescence intensity was detected in the predominant cell fraction of the NIHs culture, compared with the corresponding predominant fractions of both NIHb and the NIHs cultures. Consistent with their senescent state, both NIHv cell fractions showed lower mitochondrial fluorescence values than those detected in the other cultures analyzed here. In the NIHv cells, the decline in the functional activity of the mitochondria was linked to their elongated morphology. Similar situations have been described in other models of senescence in vitro; although, at the same time, an increase in the overall mitochondrial mass has been detected [39]. In other cases, it has been shown that mitochondria with an elongated morphology are able to express high bioenergetic capacities and the characteristic of fusing together [35]. In our experimental model, in the untreated NIHv culture, in agreement with previous reports [39], the lower level of the fluorescence of the mitochondria could be attributed, primarily, to their reduced functional activity rather than a decrease in their number. Even in the case of NIHv cells, the interpretation of the morpho-functional data of the mitochondrial compartment can be placed in a context in which opinions and some experimental evidence may appear contradictory or in any case create doubts. This is also because the onset of the senescence process could perhaps stimulate the genesis of new mitochondria but at the same time induce an acceleration of the aging processes. Indeed, increasing the number of these organelles, especially if they are not fully functional, could have the negative effect of increasing the number of cellular sites of ROS production [39] with different repercussions depending on the specific characteristics and basal redox state of the cells. In untreated NIHv cells, the lower mitochondrial content/activity was correlated with the higher level of superoxide anion production, compared with that found in cells from both NIHb and NIHs cultures. Further interpretative elements can be derived from the analysis of flow cytometry data on the mitochondrial content/activity obtained after the treatment of NIHv cultures with hydrogen peroxide. Within this culture, the predominant cell fraction, with the highest fluorescence before the treatment, was significantly depleted in its percentage incidence in favor of the fraction with the low mitochondrial content/activity. Together with the reduction in the DiOC6 fluorescence signal, this effect was associated with the increased production of the superoxide anion detected in NIHv cells after the treatment with the oxidizing agent. Thus, in NIHv cells, the high basal level of hydrogen peroxide, generated by the superoxide anion dismutation [40], would be further increased after the treatment.

In addition to the effects at the mitochondrial level, further damage could occur in other cellular components, as at least partly indicated by the increase in PI-positive NIHv cells after the treatment with the oxidizing agent. The bivariate flow cytometric analysis indicated that alterations in membrane permeability would appear to affect, in particular, cells with a lower mitochondrial content/activity. However, even in the predominant fraction of treated NIHv cells, the ratio between the mitochondrial content/activity and PI uptake tended to increase, with the most marked shift, compared with that in other cultures. Thus, the fraction of cells with a high mitochondrial content/activity shows the most drastic decrease. In NIHv cells, the mitochondrial compartment, which is already partially compromised by the pre-existing state of senescence, could be more susceptible to treatment-induced oxidative stress. This flow cytometric datum could be at least partly related to the increase in the fraction of NIHv cells with a rounded/fragmented mitochondrial morphology detected after the treatment in this culture. In the treated NIHs cells the increase was much less evident also considering that, already under basal conditions, almost 90% of the cells predominantly possess rounded/fragmented mitochondria.

Functional alterations linked to the dynamics of mitochondrial fusion and fragmentation, resulting in heterogeneous phenotypes, can also be associated with the onset and progression of tumor pathologies [41,42]. In these cases, the most frequently detected phenotype was characterized by fragmented mitochondria. This situation was associated with the high proliferative activity typical of various tumor cells but also of stem elements resistant to cellular differentiation [43]. In works performed on osteosarcoma-derived cell lines, several cell clones with an elongated or fragmented/rounded mitochondrial morphology were identified. Cells characterized by chondriome fragmentation showed better bioenergetic capacities and were more resistant to antineoplastic treatment [44]. Also, in the transformed NIHs cells of our experimental model, the homogeneous and rounded morphology of the mitochondria was associated with a higher content/activity of these organelles. From a functional point of view, this type of data would seem to contradict the view, held for several decades, that the anaerobic glycolytic metabolism prevails in transformed cells, to the detriment of mitochondrial respiration. This opinion has been critically reconsidered in the last decade, and the experimental evidence that has emerged in some works [45,46] may allow us to interpret the mitochondrial flow cytometry data of NIHs cells as indicative of their enhanced bioenergetic capacity. Based on the cellular heterogeneity that may characterize certain tumor types, some subpopulations show a metabolic dependence on mitochondrial respiration, and the ∆Ψm potential is often higher than that of normal cells [47]. This dependence would be linked to the ability to maintain the transformed phenotype and proliferative activity of the cells [43,48].

In our experimental model, in the untreated NIHs culture, even the smallest fraction of cells with the lowest mitochondrial content/activity proved substantially unable to uptake propidium iodide, thus indicating an unaltered plasma membrane state. Therefore, this fraction would consist of cells expressing specific metabolic characteristics of the mitochondria, rather than a state of cellular deterioration. If this was the case, cells with a different, more or less aerobic, metabolism could coexist in the NIHs culture. Furthermore, considering the homogeneity of the rounded morphology of the mitochondria detected in all the cells of the NIHs culture, the functional heterogeneity of the different fractions could depend on their positioning in a specific phase of the cell cycle and thus be related to the desynchronization of the culture. In other words, the fraction of NIHs cells with the lowest mitochondrial content/activity could be represented by cells that are predominantly in the S phase of the cell cycle. It has previously been shown, in other mammalian cell lines, including murine breast cancer cells, that the orientation toward the G1/S transition and the onset of DNA replication coincides with a progressive decrease in mitochondrial activity and changes in their biogenesis [36,48,49].

With regard to the relationship between the bioenergetic capacity and the extent of the ROS production, this is known to depend on various factors, including the energy status of the mitochondria, which is linked to redox reactions and their degree of efficiency along the respiratory chain [48]. In some cases, a reduced respiratory capacity has been associated with a decrease in ROS production by cells [41,43]. High ROS levels are usually a hallmark of tumor cells and are often the consequence of alterations in their metabolism. However, tumor cells manage to maintain a balance between ROS production and the activity of antioxidant systems. The modulation toward more moderate levels of ROS may be useful for the growth and maintenance of tumor cell stemness [43]. In the untreated NIHs culture, the microfluorimetric measurement of the superoxide anion showed, in the prevailing cellular fraction, a high level of production, but it was lower than that detected in senescent NIHv cells. The presence of two fractions of NIHs cells, with a different superoxide anion production, may be related to the heterogeneity of the mitochondrial content/activity values, indicating that the extent of the respiratory activity could be related to the specific ROS levels of the different cell fractions. With respect to the possible relationship between the mitochondrial morphology and ROS production, the data reported in the literature do not all agree. In some cases, especially during cell differentiation events, mitochondrial fusion has been associated with increased respiratory activity and reduced ROS. On the other hand, in several tumor cell types or cells stimulated in mitogenic activity, high ROS production was associated with the fragmentation and fission of mitochondria which assume a rounded morphology [35]. What the authors of this study described bears similarities to the mitochondrial phenotype we reported for untreated NIHs cells. The incubation of this culture with hydrogen peroxide caused a reduction in the fluorescence intensity in all NIHs cell fractions, with a different mitochondrial content/activity, already present in basal conditions. In addition, microfluorimetric analyses of the superoxide anion status after the treatment revealed an overall increase in its production. This would lead to the hypothesis that the majority of the NIHs cells, including those with a lower mitochondrial content/activity, are sensitive to additional stress conditions induced by the oxidizing agent. It can also be highlighted that, after the treatment, the percentage increase in the fraction of NIHs cells with lower mitochondrial fluorescence was correlated not only with a higher level of the superoxide anion but also with an increase in the number of PI-positive cells. Furthermore, the proportion of cells with a higher value of the ratio between the mitochondrial content/activity and PI uptake was increased. The altered state of the plasma membrane function, together with mitochondrial deficits, could be indicative of a tendency toward deteriorating cellular conditions. Therefore, the specific redox state could create the need to eliminate damaged or dysfunctional mitochondria. This could be a common requirement for cells under various biological conditions such as cell transformation or senescence. In the latter condition, it should be emphasized that the ability to dilute worn-out mitochondria by dividing them between progeny cells would be compromised as a consequence of the decline in cell division events.

### 4.3. Dynamics of the Lysosomal Compartment Before and After the Hydrogen Peroxide Treatment

Depending on changes in the proliferative activity and specific metabolic requirements of the differentiated status, cells can program their functional activities differently by finely regulating the number of mitochondria. The removal of damaged or supernumerary organelles occurs through autophagic processes involving the activity of the lysosomal compartment [50]. The negative effects of mitochondrial malfunction on energy metabolism can influence the increase in ROS, impairing the structure and function of lysosomes with repercussions on the cell degradation activity and alterations in the recycling capacity of their components [51,52]. In this way, the different mitochondrial phenotypes described in the cultures we analyzed may also influence the lysosomal compartment with respect to the occurrence of autophagic events.

In the absence of the treatment, compared with the reference NIHb culture, the NIHs culture showed cells with a significantly lower lysosomal content/activity. This feature appeared to be related to the rare accumulation of lipofuscin, as well as the lack of positivity for the SA-β-Gal reaction. The reduced extension of the lysosomal compartment of the NIHs cells was also correlated with the limited number of autophagic events which did not significantly differ from those detected in the NIHb culture. In analogy to the NIHs culture, previous work showed that the lysosomal compartment of NIH/3T3 cells, after the transformation induced by the SV40 virus, was modified with a reduction in the number and total mass of lysosomes per cell [53]. Other experiments performed on the same experimental model indicated an increase in the volume of the lysosomal compartment, dense bodies, and autophagic vacuoles depending on the conditions of cell confluence or the absence of serum in the culture medium [54]. These findings may support evidence that the modulation of the proliferative activity may affect the state of the lysosomal compartment. In poorly proliferating NIHv cells in the absence of the treatment, the examination of microfluorometric frequency curves indicated the presence of two distinct cellular fractions: the prevalent one with a high lysosomal content/activity and the second fraction with a lower percentage incidence and a lower lysosomal content/activity. In the prevalent fraction of the NIHv culture, the measured lysosomal content/activity was the highest compared with that of all cell fractions in the other cultures tested. These features of the lysosomal compartment appeared to be correlated with the morphological aspects, detected after the staining with Acridine Orange, which showed that the red labeling was often concentrated in some areas of the cytoplasm, indicating the higher incidence of autophagic processes. This phenomenon may also be at least partly related to the presence of aggregates of both lysosomal fluorescence (LTR) and lipofuscin inclusion autofluorescence. Autodegradative activities, which are frequently found in other senescent cultures, were associated with drastic changes in lysosomes, in terms of the number and size [50,51,52]. In untreated NIHv cells, degradation events appear to be at least partially ineffective, as would be shown by the higher degree of lipofuscin accumulations. These inclusions would result from undigested material, due to incomplete self-digesting processes. The NIHv cell fraction with a low lysosomal content/activity, detected by the microfluorimetric analysis, and that with a low mitochondrial content/activity, deduced from the relative dot plot examination, showed almost coinciding percentage incidences. Therefore, the same cells could show deficits in both compartments. In other cellular models, the impairment of lysosomal activity, caused by the increased ROS produced by dysfunctional mitochondria, was expressed morphologically, with the formation of lysosomal vacuolations [51]. Overall, what emerges from comparing cultures of NIHs and NIHv under basal conditions indicates how the lysosomal content/activity may be regulated differently depending on the cytokinetic characteristics and the prevalence of transformed or senescent phenotypes. Comparing these two cultures, the lower lysosomal content/activity of the transformed NIHs cells correlated with a higher mitochondrial content/activity and a lower level of superoxide anion production. In contrast, in senescent NIHv cells, these same parameters showed an opposite trend. In our opinion, these results would indicate, even in our experimental model, the existence of a kind of interaction network between the two types of organelles. Lysosomes would play a key role not only because they are implicated in mitophagic processes but, as shown in other works, they could also be involved in the regulation of mitochondrial fission [55]. As previously reported, the balance between mitochondrial fission and fusion may have implications for cell cycle control [35]. On the other hand, in all the cultures analyzed here, the cytostatic effect induced by hydrogen peroxide, although of a different magnitude, was reflected in the tendency toward an increased lysosomal content/activity. However, the microfluorometric patterns revealed several different aspects, in quantitative terms and in relation to the dynamics of the cell fractions in the cultures. After the incubation of NIHb reference cells with the oxidizing agent, there was a limited increase in the lysosomal fluorescence and lipofuscin inclusions. The SA-β-galactosidase labeling, already negative under basal conditions, was also slightly changed. In contrast, in NIHs cells, in which hydrogen peroxide induced the most significant decrease in proliferative activity, the greatest increase in the lysosomal content/activity was detected compared with that under the basal conditions. In this culture, the quantitative data obtained after processing fluorescence microscopy images revealed the presence of aggregates of lysosome/acidic vesicles that tended to co-localize with autofluorescent cytoplasmic inclusions of lipofuscin, which also increased after the treatment. The increase in the lysosomal content/activity detected in NIHs cells after the hydrogen peroxide treatment could be at least partly related to the overall reduction in the mitochondrial content/activity and the percentage increase in the fraction of cells with a lower content/activity of these organelles. This type of interconnection would result from the need to eliminate less efficient mitochondria. Their removal would be linked to the activation of autophagic processes, which increase after the treatment, and which would perhaps be aimed at decreasing the level of ROS increased due to the mitochondrial malfunction. Compared with those under basal conditions, the higher level of autophagic processes, in treated NIHs cells, was correlated not only with an increase in the lysosomal content/activity but also with an increase in the number of fluorescent aggregates of both lysosomes themselves and lipofuscin inclusions. These aspects were also found to be concomitant with the appearance of positivity in the SA-β-galactosidase reaction. Similar lysosomal dynamics, which are also related to the inefficiency of the mitochondrial compartment, could occur in the populations of NIHv cultures, even under basal conditions, whereas in NIHs cells, they would be linked to senescence conditions, induced by the treatment.

In poorly proliferating NIHv cells, the bimodal pattern of the lysosomal fluorescence intensity distribution, detected in the absence of the treatment, changed to a unimodal distribution after the incubation with hydrogen peroxide and showed an overall mean increase in the signal intensity. The achievement of the greater homogeneity of the lysosomal compartment could be due to treatment-induced stress conditions that would also involve those NIHv cells that, in the absence of treatment, had not yet fully manifested their senescence state. Thus, a greater proportion of NIHv cells would have a more adequate lysosomal compartment, which is necessary to increase turnover processes, based on the activation of autophagic events. Therefore, in both NIHs and NIHv cultures, the regulation of the equilibrium that leads cells to achieve a specific lysosome content/activity could also be related to the extent of the degradation processes involving these organelles. The accumulation of lipofuscin inclusions, detected under basal conditions in NIHv cells and after the treatment with hydrogen peroxide in NIHs cells, may be related to the formation of lipid peroxides caused by the action of ROS primarily on the unsaturated lipids of cell membranes. The products of their degradation, combined with other substances, lead to the formation of autofluorescent lipofuscin pigments which tend to accumulate in lysosomes [56].

In perspective, it would be useful to investigate to what extent the partial co-localization between the lipofuscin autofluorescence and lysosome labeling may also be affected by the leakage of these pigments from these organelles. Their membrane may be damaged by senescence-related conditions, including the peroxidation of the lipid component.

The autophagic activity detected by means of the Acridine Orange fluorochromization showed some differences when the three cultures were compared under basal conditions. Autodegradative processes were detected mainly in the NIHv cultures and less frequently in the NIHb and NIHs cultures. In NIHb cells, the hydrogen peroxide treatment induced a limited increase in the number of autophagic events, which, on the other hand, appeared to be more prominent in the NIHs culture and much greater in the NIHv culture. In both NIHs and NIHv cultures, the increase in autophagy events was correlated with an increase in lipofuscin inclusions at the cytoplasmic level. In particular, for NIHs and NIHv cells, the quantitative data for fluorescent lipofuscin aggregates (LPAI) and the red areas labeled with Acridine Orange (MAI) revealed similar trends.

If this relationship is valid, it could indicate at least some inefficiency of autodigestive processes. Different levels of lysosomal impairment would lead to a variable cytoplasmic accumulation of autofluorescent undigested material. Under different experimental conditions, the morphological and microfluorometric evaluation of the lipofuscin autofluorescence provided information that, in our opinion, is consistent with the measurements of the lysosome content/activity in the three cultures examined.

After the incubation of NIHs cells with hydrogen peroxide, the autofluorescence of lipofuscin, which was reduced under basal conditions, increased, reaching values, both in the intensity and percentage incidence, which were close to those of NIHv cells in the absence of the treatment. The trend of this biomarker, together with the acquisition of SA-β-Gal positivity, would testify to the state of senescence acquired by the NIHs cells after the treatment. In NIHv cells, which are already senescent under basal conditions, the increase in the lipofuscin autofluorescence, induced by the treatment with the oxidizing agent, was, however, not correlated with substantial changes in the labeling of the SA-β Gal activity. Thus, specific responses to stress conditions induced by the treatment would be influenced by variations in the redox status related to the different cellular phenotypes present in NIHs and NIHv cultures, resulting in the induction or modulation of senescence, respectively. In the cultures examined, the regulation of the balance between the mitochondrial and lysosomal compartments could lead to variable effects on different cellular functions, including proliferative activity.

## 5. Conclusions

In conclusion, the results obtained in this work may offer opportunities to discuss some aspects that characterize the complexity of tumors in relation to the presence of heterogeneous cell subpopulations with different proliferative activities and features attributable to pro-senescent and senescent phenotypes. The variable responses to the hydrogen peroxide treatment, in addition to depending on the different cytokinetic dynamics of the cultures examined, appeared to be related to the specific redox state and functional properties of the cells. In this context, the activities of the mitochondria/lysosomes axis could be involved in the regulation of cellular transformation and senescence processes. The different changes in the redox state of NIHs and NIHv cells could be the result of the balance produced by the contribution of endogenous ROS and those produced due to the additional oxidative stress induced by the hydrogen peroxide treatment. In perspective, further studies in this context could contribute not only to a better understanding of the biology of the cellular processes described but also to consolidating the basis for the development of alternative therapeutic strategies aimed at controlling cellular senescence and its induction in tumor cells.

## Figures and Tables

**Table 1:** The size of lipofuscin aggregates and the co-localization with the lysosome compartment.

Cell Types and Treatment	Lysosome Aggregate Index (LYAI) (%)	Lipofuscin Aggregate Index (LPAI) (%)	Lipofuscin/Lysosome Fluorescence Co-Localization Index (%)
NIHb (−H_2_O_2_)	0.10 ± 0.00	2.55 ± 0.39	0.08 ± 0.00
NIHb (+H_2_O_2_)	1.35 ± 0.34	3.05 ± 0.45	1.93 ± 0.30
NIHs (−H_2_O_2_)	5.65 ± 0.41	2.97 ± 0.51	4.42 ± 0.70
NIHs (+H_2_O_2_)	12.15 ± 0.95	7.15 ± 1.22	22.25 ± 1.51
NIHv (−H_2_O_2_)	18.61 ± 1.35	10.69 ± 1.27	26.40 ± 1.62
NIHv (+H_2_O_2_)	26.23 ± 2.12	17.12 ± 1.28	31.17 ± 2.03

**Table 2:** Percentage incidence and Mean Autophagic Index (MAI) before and after hydrogen peroxide treatment.

	Untreated		Hydrogen Peroxide	
Cell Culture	Percentage	MAI	Percentage	MAI
NIHb	4.91 ± 0.44	2.70 ± 0.25	7.76 ± 0.72	3.31 ± 0.23 #
NIHs	6.05 ± 0.48 *	3.29 ± 0.39 *	15.49 ± 1.17	7.01 ± 1.56
NIHv	23.21 ± 1.52	8.26 ± 0.53	37.27 ± 2.39	18.52 ± 1.37

The mean values reported refer to the quantification of parameters related to autophagic events detected, after the fluorochromization with Acridine Orange, under different experimental conditions. The percentages indicate the cells considered positive on the basis of the determination of the Mean Autophagic Index (MAI). The positivity threshold, equal to 2.30, was established taking into account the MAI value measured most frequently in NIHb culture cells in the absence of the hydrogen peroxide treatment (for details, see Section 2). To calculate the reported values, at least 200 cells were analyzed for each culture under different experimental conditions. The numerical values represent the means ± SE of triplicate determinations (*n* = 3). Asterisks (*) indicate values for untreated NIHs and NIHv cells that are not significantly different (*p* > 0.05 after Student’s *t*-test) from those of untreated NIHb cells. Hashtags (#) indicate values that are not significantly different (*p* > 0.05 after Student’s *t*-test) when comparing, for the same culture, the conditions before and after the treatment with hydrogen peroxide. All other mean values are significantly different (*p* < 0.05 after Student’s *t*-test).

**Table 3:** A data summary of the main parameters analyzed.

Cell Types and Treatment	Proliferative Activity (% S Phases)	Mitochondrial Content/ Activity (Fluorescence Intensity)	Superoxide Anion Detection (Fluorescence Intensity)	Lysosomal Content/Activity (Fluorescence Intensity)	Lipofuscin Autofluorescence Intensity	SA-β-Gal Positive Cells (%)	Mean Autophagic Index
NIHb − H_2_O_2_	22.2 ± 1.0	166.4 ± 3.6	98.8 ± 1.7	173.6 ± 3.8	0.0 ± 0.0	1.0 ± 0.0	2.7 ± 0.3
NIHb + H_2_O_2_	17.3 ± 0.9	153.1 ± 3.8	103.3 ± 2.1	179.9 ± 4.0	84.7 ± 2.1	2.0 ± 0.0	3.3 ± 0.2
NIHs − H_2_O_2_	34.2 ± 0.8	190.2 ± 4.1	138.4 ± 2.4	155.5 ± 2.8	108.5 ± 2.4	1.0 ± 0.0	6.1 ± 0.5
NIHs + H_2_O_2_	15.4 ± 0.8	164.2 ± 4.2	157.4 ± 2.9	183.5 ± 3.2	123.3 ± 2.7	86.4 ± 3.1	7.0 ± 1.6
NIHv − H_2_O_2_	9.2 ± 0.7	149.2 ± 3.4	145.9 ± 2.0	137.9 ± 2.1	124.5 ± 2.3	91.2 ± 2.1	23.2 ± 1.5
NIHv + H_2_O_2_	5.8 ± 0.4	130.3 ± 2.7	163.3 ± 2.8	209.2 ± 3.6	139.1 ± 2.8	96.30 ± 2.0	18.5 ± 1.4

## Data Availability

All data generated or analyzed during this study are included in this published article.
